# CoVEffect: interactive system for mining the effects of SARS-CoV-2 mutations and variants based on deep learning

**DOI:** 10.1093/gigascience/giad036

**Published:** 2023-05-23

**Authors:** Giuseppe Serna García, Ruba Al Khalaf, Francesco Invernici, Stefano Ceri, Anna Bernasconi

**Affiliations:** Dipartimento di Informazione, Elettronica e Bioingegneria, 20133 Milano Country: Italy, Italy; Dipartimento di Informazione, Elettronica e Bioingegneria, 20133 Milano Country: Italy, Italy; Dipartimento di Informazione, Elettronica e Bioingegneria, 20133 Milano Country: Italy, Italy; Dipartimento di Informazione, Elettronica e Bioingegneria, 20133 Milano Country: Italy, Italy; Dipartimento di Informazione, Elettronica e Bioingegneria, 20133 Milano Country: Italy, Italy

**Keywords:** deep learning, language models, machine learning interpretability, CORD-19 dataset, SARS-CoV-2, viral variants, viral mutations, web interface

## Abstract

**Background:**

Literature about SARS-CoV-2 widely discusses the effects of variations that have spread in the past 3 years. Such information is dispersed in the texts of several research articles, hindering the possibility of practically integrating it with related datasets (e.g., millions of SARS-CoV-2 sequences available to the community). We aim to fill this gap, by mining literature abstracts to extract—for each variant/mutation—its related effects (in epidemiological, immunological, clinical, or viral kinetics terms) with labeled higher/lower levels in relation to the nonmutated virus.

**Results:**

The proposed framework comprises (i) the provisioning of abstracts from a COVID-19–related big data corpus (CORD-19) and (ii) the identification of mutation/variant effects in abstracts using a GPT2-based prediction model. The above techniques enable the prediction of mutations/variants with their effects and levels in 2 distinct scenarios: (i) the batch annotation of the most relevant CORD-19 abstracts and (ii) the on-demand annotation of any user-selected CORD-19 abstract through the CoVEffect web application (http://gmql.eu/coveffect), which assists expert users with semiautomated data labeling. On the interface, users can inspect the predictions and correct them; user inputs can then extend the training dataset used by the prediction model. Our prototype model was trained through a carefully designed process, using a minimal and highly diversified pool of samples.

**Conclusions:**

The CoVEffect interface serves for the assisted annotation of abstracts, allowing the download of curated datasets for further use in data integration or analysis pipelines. The overall framework can be adapted to resolve similar unstructured-to-structured text translation tasks, which are typical of biomedical domains.

## Introduction

The COVID-19 pandemic has made SARS-CoV-2 one of the most studied viruses in the world, with research on its variation, spread, and impacts on the host immune system. At the start of 2020, it was estimated that 200,000 coronavirus-related journal articles and preprints would be published by the end of the year [1]. As of today, about 3 years since the beginning of the pandemic, more than 1 million articles have become available.

This wide COVID-19–related literature is still largely unexplored but can be employed for data and text analysis. Most COVID-19 research outputs have been gathered within the COVID-19 Open Research Dataset (CORD-19 [2]) by the Allen Institute. The corpus includes preprints and papers from Semantic Scholar up to mid-2022, sourced from PubMedCentral, PubMed, the World Health Organization’s Covid-19 Database, and the preprint servers bioRxiv, medRxiv, and arXiv.

In parallel, there has been a worldwide spread of open data representing SARS-CoV-2 sequences (through the data sources GISAID [3], GenBank [4] and COG-UK [5]), gathered on repositories by public and private institutes. The study of viral sequences has addressed several research questions related to the epidemiology and immunology aspects of the viral spread [6–8]. Much attention has also been dedicated to identifying amino acid–level mutations (or groups of them—coordinated within variants) that lead to particular changes in the behavior of the virus and its ability to establish infections—when compared to the wild type [9–11]. Note that, currently, it is hard to integrate data about sequences (with associated mutations) with information about variation effects, as the latter is not available in structured formats.

Structured information can be retrieved resorting to Natural Language Processing (NLP) techniques. NLP models usually require a considerable quantity of training data to learn their tasks. However, recent breakthroughs with deep learning models such as the Generative Pretrained Transformer (e.g., GPT2 [12]) allowed the design of multitask learners that use fewer data than classic supervised machine learning techniques.

In this work, we use GPT2 to learn tuples that contain a SARS-CoV-2 variation, its effect and level, starting from CORD-19 abstracts. The model is trained on a small dataset that we carefully fabricated, as no such ready-to-use dataset was available. As our system enables expert users to provide more input annotations, it is preferable to use a model that dynamically and efficiently learns how to handle new annotations over time; in parallel, it is desirable to augment the training dataset in a continuous manner. To allow for this, we use a semiautomated data labeling system, which employs the predictive model to assist the human labeler, combining manual annotations with automatic tuples extraction. The model is used to recommend labels and automate basic functions in a labeling interface. The user can decide when to employ the generated labeled data for augmenting the training dataset and retraining the model. A user-friendly web interface CoVEffect allows expert users to annotate abstracts with variation effects without requiring any programming or data management knowledge.

## Related Work

Currently, the task of recognizing mutations and variants’ effects needs to be performed by hand. There are a very few resources that provide this kind of information; when this is the case, they are exclusively manually curated. FaviCoV and ESC [13, 14], respectively, store SARS-CoV-2 genetic mutations that are functionally relevant and are associated with immune escape. The antigenic role of amino acid replacements in the context of the human immune response is also the focus of the COG-UK Mutation Explorer [15], while a list from the World Health Organization (WHO) concentrates on specific replacements that characterize variants [16]. Torrens-Fontanals et al.[17] report on how variation impacts can be predicted. Online resources such as CoVariants [18], European Centre for Disease Prevention and Control [19], WHO [20], and Centers for Disease Control and Prevention (CDC) [21] explain variants’ effects, commenting on how they are reported in the literature. We previously made extensive curation of effects stored in CoV2K [22], a knowledge base of data and knowledge about SARS-CoV-2; our cumbersome manual curation approach had quickly become unfeasible, prompting us to explore alternative solutions.

Several NLP techniques have been used and adapted to bioinformatics-relevant problems, as reported in surveys such as [23] or [24]. Research applications concerned omics (e.g., prediction of protein classification/structure [25], motifs [26], or drugs to be developed [27]) and biomedical imaging/signal processing [28].

Regarding biomedical text extraction, a wealth of studies is focused on clinical NLP, regarding electronic health records and clinical notes [29–31]. For extracting phenotype–genotype relationships, Singhal et al. [32] proposed a 3-step pipeline that (i) recognizes 3 different kinds of entities (mutations, diseases, genes) with entity-specific tools of PubTator [33], (ii) links mutations with diseases using a Machine Learning (ML) binary classifier [34], and (iii) interprets mutations in the context of specific genes.

A very recent tool called ViMRT [35] employs ad hoc optimized rules and regular expressions for the extraction of viral mutations; a whole infrastructure is built with this sole purpose, demonstrating the complexity of the task, whose resolution remains largely uncovered.

Instead, the most recent approaches to biomedical text extraction tasks have employed transformer-based techniques, as reviewed in [36] and [37–39]; they report that current works are mainly focused on connections between entities [40, 41]. Very few works addressed results’ explainability combined with transformers in this domain [42, 43].

In our past work [44, 45], we employed deep learning transformer-based techniques for NLP to infer attributes from Gene Expression Omnibus [46] experiment metadata, formulating the problem as a translation task. Cannizzaro et al. [44] and Serna Garcia et al. [45] achieved the result of translating Gene Expression Omnibus experiment descriptions into key:value pairs (e.g., cell line:K562, disease:myeloid leukemia, assembly:hg19, assay:Chip-Seq, target:H3K9me3).

CoVEffect stems from this thread of works, but it is carefully adapted to solve a more complex task: that of predicting a series of tuples from SARS-CoV-2–related abstracts where we consider a variation, its effect, and the change of its level. Each of the currently available systems supports only one user-driven annotation [47], predictions of single independent annotations with ontological terms [48], or biomedical general-purpose triplets based on existing knowledge graphs [49], especially targeted to protein–protein interactions [50]. These correspond to different tasks than the one performed by CoVEffect, and the described approaches do not allow for online modifications of the training dataset. Our purpose is closer in spirit to the one targeted in Mahajan et al. [51]; however, their work is focused on clinical aspects (text is extracted from electronic health records instead of research abstracts) and is not supported by a user-oriented interface.

All in all, to the best of our knowledge, CoVEffect is one of the first transformer-based approaches applied to biomedical tasks, combined with explainability approaches.

## Materials and Methods

**Figure 1:**
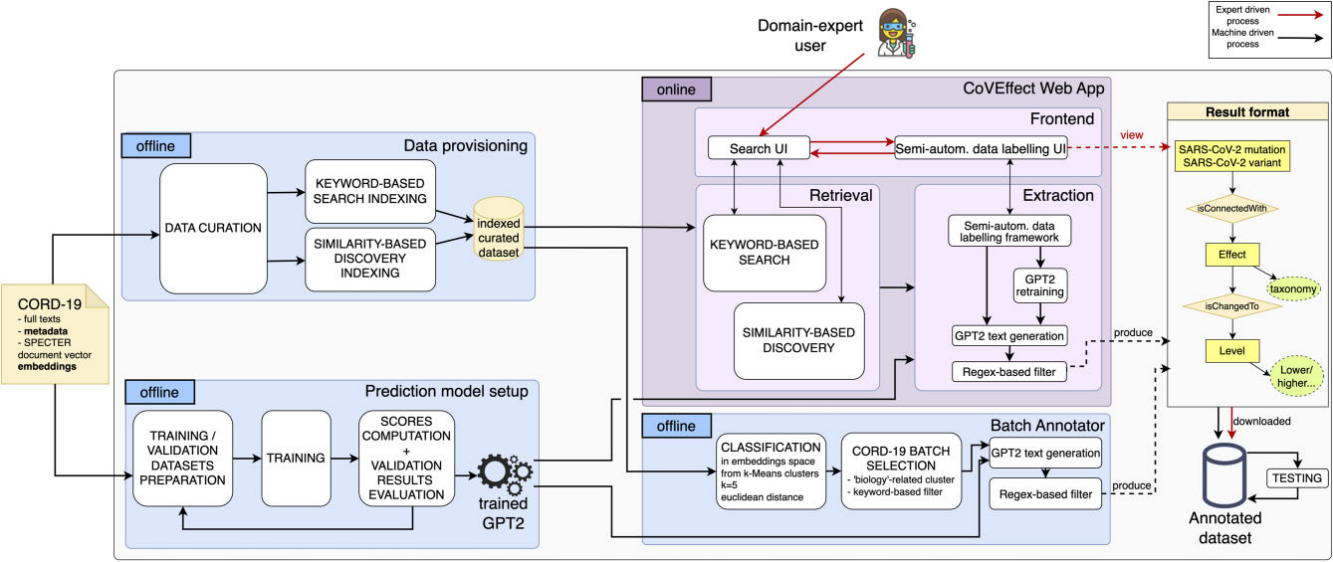
CoVEffect framework overview.

Figure 1 captures the high-level architecture of the whole framework. As our input, we consider the wealth of information contained in the CORD-19 dataset. From the data corpus, we extract only abstracts that reach sufficient quality standards and provide essential metadata.

Two offline processes exploit the dataset: (i) *data provisioning*, where we perform data curation and prepare a dataset that supports indexed keyword-based search and similarity-based search, and (ii) *prediction model setup*, where we manually craft a dataset, use it for training the model, check its performances (through a validation dataset), and evaluate the need to change or augment the initial training dataset.

The artifacts produced by these 2 processes are the indexed curated dataset of CORD-19 abstracts and the trained prediction model. They feed 2 possible modes of use, sharing standardized output formats:

- an offline *Batch Annotator*, which provides annotated data for a selection of 7,230 relevant abstracts from the CORD-19 corpus, and
- an interactive online *Web Application* employed by expert users to annotate samples and inspect predicted annotations.

### Data provisioning

From the latest and final CORD-19 release (issued in June 2022), we collected *metadata.csv*, a table with metadata of all papers, and *cord_19_embeddings.tar.gz*, a collection of precomputed SPECTER [52] document embeddings for each paper. The data provisioning pipeline aims to produce a curated set of abstracts (equipped with metadata) to support the activities of the learning framework.

#### Data curation

As described by Wang et al. [2], the CORD-19 dataset gathers COVID-19–related papers from several sources. In this dataset, papers are already harmonized and de-duplicated: in the metadata table, each *cord_uid* represents a cluster of papers with colliding identifiers, such as *DOI* or *arxiv_id*. For our system, we extracted a portion of the original CORD-19 dataset: we kept only 1 record for each paper, thereby avoiding duplicated entries and easing the annotation user experience. To this end, we designed a reconciliation step: for each cluster, we favored the entry with the longest abstract and promoted values from other members of the cluster to fill in the missing information; then, we removed the other members of the cluster, obtaining only 1 entry for each paper. We also removed those papers for which an abstract was not available. Additionally, we used *langdetect* [53]—a language detection library ported from Google’s *language-detection—*to detect the language of the abstracts and filtered out the papers not written in English.

#### Abstract retrieval

The curated dataset has been indexed to support search on the paper abstracts. Such a step is functional to the retrieval task of the learning system, where the user searches abstracts that are of interest. For the purpose, we built a search engine leveraging 2 existing libraries.

- The *keyword-based search* is based on *Whoosh* [54], a full-text indexing and searching library, to let users search the abstracts using combinations of keywords.
- The *similarity-based discovery* is based on *Annoy* [55], an approximate nearest-neighbor search library, to let the users discover abstracts similar to those already selected. These recommendations are computed by exploiting the SPECTER embeddings of the papers, which are document-level vector representations originated from citation-based transformers. For our purpose, we dramatically reduced the dimensionality of the vector space from 768 to 100. The 100 dimensions were selected by means of a principal component analysis, resulting in a representation with an explained variance ratio of 74%. In line with the recommendation task overviewed in [52], we chose cosine similarity as a similarity metric among papers by setting the distance parameter of the AnnoyIndex to “angular.”

### Language model and task design

#### Model

In this work, we favored text-generative transformer models over BERT-like models [56] because of their ability to perform multitask learning [12] and to easily adapt to new tasks. Indeed, text-generative models formulate multitask learning as a conditional distribution *P*(*output*|*input,task*), where the task to be performed can be easily expressed in the form of text. We also make a distinction between general and domain-specific pretrained models. General models are usually pretrained with large datasets aiming to be as general as possible (e.g., BookCorpus [57] and English Wikipedia). Domain-specific models, instead, are further pretrained in order to fit a particular application (e.g., medicine, biology). In our case, the specific domain knowledge is represented by the CORD-19 dataset [2]. In the past years, several new generative models have been proposed (e.g., T5, BART, GTP3, BLOOM). These models achieved increasingly better performances, mostly by increasing the size of the model parameters and the size of the pretraining datasets. As a trade-off, bigger models are significantly slower.

In our work, the model is also used in an interactive way (with a domain expert), and thus we preferred smaller models to large models. Considering all these aspects, we opted for a domain-specific version of a gpt2-small model available on the huggingface model hub [58]; it represents a reasonable compromise between model size and performances in a very specific domain. We propose it as a baseline for future works that could make use of our dataset.

#### Target data format

Abstracts are annotated by recognizing structured tuples of the form ⟨*type,entity,effect,level*⟩. Possible *types* are “mutation” and “variant.” With *mutation*, we refer to amino acid changes within specific proteins, occurring in a position where a reference residue has been changed into an alternative residue. These changes correspond to nonsynonymous nucleotide mutations; we do not consider synonymous nucleotide mutations, as they typically do not influence the protein functionalities. In this work, we focus on substitutions, leaving aside insertions and deletions as they would require substantial additional training due to their very heterogeneous formulations. With *variant*, we denote forms of the SARS-CoV-2 that are considerably different from the original wild-type [59], as they accumulated a set of amino acid changes that characterize their phenotypic characteristics [60]. Variants are typically associated with a name to easily address them.

In our tuples, *entities* are the names of mutations (e.g., Spike_N501Y or NSP12_P323L) or of variants—for example, Alpha, Delta, Omicron (as named by WHO [20]) or B.1.1.7, B.1.617, B.1.519 (as named by Pangolin [61]).

*Effects* are chosen from a taxonomy, that is, a controlled vocabulary of terms, including, for example, transmissibility, disease severity, resistance to antiviral drugs, or change in the protein kinetics (flexibility or stability properties). We previously proposed an initial version of this vocabulary [22, 62], which has now evolved into a complete list of effects organized by category (“epidemiology,” “immunology,” “viral kinetics and dynamics,” or “diagnosis, prevention, and treatments”). The full list can be found the AdditionalFile1-effects-taxonomy [63].

Finally, each effect has an associated *level*, that is, higher, lower, unaffected, undefined, or no evidence (see AdditionalFile2levels-taxonomy [63] for detailed definitions).

#### Task

The macro-task performed by our prediction model is a text-to-table task, translating a full paper abstract into a table of tuples, each one with the fields described above. Each tuple is composed itself by solving 3 subtasks:

- entity extraction of *mutations/variants* (from which also the *type* is inferred);
- classification of *effects*; and
- classification of *levels*.

Tasks (ii) and (iii) are classic classification tasks, targeting a known set of values. Instead, the entity extraction task (i) is more complex than a classical Named-Entity-Recognition (NER) task: we extract mutations and variants with an associated effect and corresponding level. The complexity of this macro-task increases also because the number of tuples of the table output for each abstract is not fixed *a priori*. Instead, it depends on the number of extracted entities and on the number of effects exhibited by the entities. Text-generative models allow to fine-tune a single model that is able to perform this macro-task.

**Figure 2:**
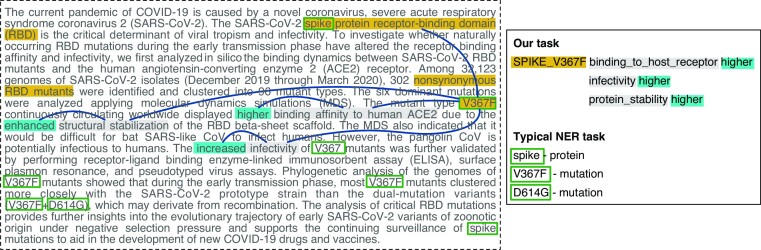
Difference between tasks resolved by an NER approach (only recognizing entities from a text excerpt) and our translation-based approach (targeting entities with connected effects and levels). The abstract excerpt is extracted from a paper by Ou et al. in the *Journal of Virology* [64]. Information used to form our tuples is connected through blue lines. Yellow identifies information on *type* and *entity*, gray on *effect*, and blue on *level*. Green rectangles identify the typical entity extraction performed by an NER approach.

Figure 2 illustrates the working principle of our prediction task on a real abstract [64]. Three different tuples are recognized in the text, all referring to the Spike V367F mutation, but predicating on different effects with higher levels. Note that the information about the protein on which the mutation occurs is positioned in a part of the text that is far apart from the signature of the mutation. In the figure, we can also appreciate the difference between the predictions obtained by our approach versus the ones that a typical NER task could obtain.

#### RegEx-based prediction filtering

A common issue for text-generative models is the instability of the generated text (i.e., these models tend to repeat words or to generate meaningless words). To mitigate this effect, we make use of a filter based on regular expressions that only allows outputs of the model corresponding to predefined legal values. The RegEx filter is applied after the extraction of mutations and variants to include only predictions that follow these patterns:

- Mutations: ^([A-Z0-9]+_)[A-Z]\d{1,4}[A-Z]$
- Variants: ^([A-Z]{1,2}\.[0–9]{1,3})(\.[0–9]{1,3}){,2}$

### Prediction model setup

The previously described task is more complex than a classical NER task, as it requires to connect different linked information. In biomedical literature, training datasets for supervised learning are typically available for general biomedical terms [38], which are of no use for our purpose; therefore, we prepared our own training dataset. This operation requires a costly manual curation, operated by highly expert users. This is an inevitable effort to handle data scarcity, analyzed in [65] in general terms, becoming even more relevant in biomedical fields [66, 67]. To minimize such effort in our case, we implemented a process that supports the building of small high-quality training datasets.

**Figure 3:**
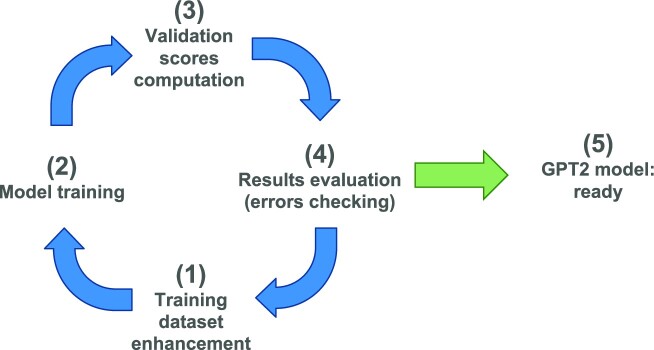
Iterative process for the prediction model setup.

We started with a small number of initial abstracts (corresponding to a first set of 30 papers). Using this seed, we used an iterative process of 4 steps (represented in Fig. 3):

1. Training dataset enhancement. Except for the first round (30 abstracts), at each iteration, we include (typically 5) new abstracts, allowing stronger training on insufficiently represented cases.
2. Model training. This procedure includes parameter tuning and possible changes based on previously obtained results.
3. Validation scores computation. The model prediction performances are evaluated on a validation dataset of 50 papers, carefully chosen to be as representative as possible of the problem at hand. By comparing expert-provided annotations and predicted annotations on the validation dataset, we compute performance scores.
4. Evaluation of results and errors. The obtained scores are considered; the iteration is repeated until satisfactory scores are obtained.

#### Training dataset preparation

The set of abstracts used for initial training was built by following a number of criteria:

- priority was given to published articles over preprints, excluding papers that duplicated the same research;
- priority was given to simple abstracts over abstracts with numerous and complex annotations;
- a wide selection of mutations (from different proteins) and variants (both WHO- and Pangolin-based names) was employed;
- abstracts involving mutations of insertion/deletion types were excluded at this stage, due to their highly heterogeneous representations;
- abstracts associating effects to groups of mutations (rather than to a single mutation or variant) were also excluded not to overly complicate the prediction task; and
- no effect of our taxonomy (see AdditionalFile1-effects-taxonomy [63]) was underrepresented in the dataset.

Table 1 shows comprehensive counts of abstracts containing information on each effect of our taxonomy, both for the training and the validation datasets; AdditionalFile3-training_dataset_target [63] contains the manual annotations associated with the 221 abstracts selected for training (after several iterations on the process shown in Fig. 3).

**Table 1:** Number of abstracts representing each effect in the validation and train datasets

Category	Effect	# training abs	# valid. abs	# training tuples	# valid. tuples
Viral kin. and dyn.	protein_flexibility	8	3	16	11
	protein_stability	29	3	47	9
	host–virus interactions	5	1	12	1
	binding_to_host_receptor	45	7	94	10
	binding_to_antibodies	16	3	30	3
	viral_load	27	7	30	9
	viral_incubation_period	8	1	9	1
	viral_replication	16	2	22	2
	viral_fitness	14	4	21	10
	intermolecular_interactions	20	2	0	2
	protein_functioning	19	2	32	5
	protein_conformational_optimization	28	4	60	6
	entry_efficiency	9	1	14	1
Immunology	sensitivity_to_antibodies	18	9	24	20
	sensitivity_to_convalescent_sera	20	6	32	10
	sensitivity_to_vaccinated_sera	20	6	35	11
	immune_escape	35	11	69	21
Epidemiology	viral_transmission	66	18	95	33
	infectivity	44	13	65	24
	viral_virulence	9	3	22	5
	disease_severity	32	8	62	15
	risk_of_hospitalization	10	7	26	10
	risk_of_reinfection	11	3	11	7
	fatality_rate	20	9	36	12
	infection_duration	7	1	9	1
Diag/Prev/Treatm.	effectiveness_of_available_diagnostics	13	1	23	1
	effectiveness_of_available_vaccines	37	13	50	29
	effectiveness_of_available_antiviral_drugs	23	6	50	10
	ct_value	12	2	14	2
*No relevant tuples found*		9	1	—	—
**Distinct abstracts/tuples**		**221**	**50**	**1,051**	**282**

#### Model training

In the iterative process, the “model training” phase is run in 2 different modes: (i) short-cycle training and (ii) long-cycle training:

- *Long-cycle training* employs the whole training dataset collected thus far to train the pretrained gpt2-model [58] all at once. It is triggered when a relevant number of annotations (60) have been collected. A manual inspection of the learning curves is conducted to perform appropriate hyperparameter tuning; the number of epochs is determined by performing an early stopping (using the validation set). When the training concludes, we generate a model freezed version (checkpoint) to be used in the following phases (validation and errors checking).
- *Short-cycle training* is triggered when 5 new abstracts are added to the training set, aiming to update the system as soon as the new annotations are available. Here, no hyperparameters are used, and the learning rate is set to half of the long-cycle training one, in order to avoid overfitting.

In both modes, the used maximum token length is 1,000, and AdamW [68] is used as the optimizer. The final model was trained for 12 epochs with a learning rate of 1*e* − 5 and a batch size of 1.

#### Scores computation

The target annotations performed by our expert researchers are available at AdditionalFile4validation_dataset_target [63] and are supported by the text document AdditionalFile5-validation_dataset_highlighted [63], where we highlighted in yellow information used by experts to inform the annotation process and derive the target tuples.

We compare the expert annotations with the predictions of the model (see AdditionalFile6-validation_dataset_prediction). Six scores are computed for 2 different scenarios: (i) we evaluate entities, effects, and levels separately (note that types are not included as they can easily be inferred from the syntax of the entity), and (ii) we evaluate whole tuples, including an entity with its linked effect and linked level. By comparing the target tuples—from zero to many in each abstract—with the predicted tuples, we assess the number of true positives, false positives, and false negatives. Based on these observations, we compute the *accuracy*, *precision*, and *F1 score* of each abstract. Then, we obtain 2 aggregate scores as a simple average of the single-abstract scores (i.e., each abstract contributes equally) and a weighted average (i.e., each abstract contributes proportionally to the number of contained target tuples).

In Table 2, we show the results on the 50 papers of the validation dataset. Evaluating fields separately and using a normal average, the trained model reached 0.79 F1 score on mutation/variants, 0.63 on the effects (independently on their link to an existing entity), and 0.76 on their levels (independently on their link to an existing entity or effect). Especially for entities and effects, precision was higher than recall, indicating that the model performed well in identifying actual positives. Specifically, out of all the predicted *entities*, almost 88% were actually present in the abstracts; out of all *effects*, 74% were actually present; and out of all *levels*, about 76% were actually present. Recall was slightly lower for entities and effects, indicating that the model missed some target information in the abstract. Specifically, recall was about 77% for *entities*, meaning that about 23% of actual entities were not recognized in abstracts; similarly, about 38% *effects* were not recognized and 24% *levels* were not recognized. Performances computed with the weighted average are generally lower, suggesting that simple abstracts (with few annotations) are the ones that contribute to improving the scores.

**Table 2:** Validation set results (run to set up the prediction model)

Task	Measure	F1 score	Precision	Recall
**Entity**	Average Weighted average	0.791 0.668	0.878 0.806	0.766 0.613
**Effect**	Average Weighted average	0.626 0.561	0.741 0.716	0.617 0.531
**Level**	Average Weighted average	0.762 0.702	0.763 0.705	0.761 0.701
**Whole tuple**	Average Weighted average	0.463 0.354	0.588 0.519	0.441 0.300

Finally, performances are considerably lower for the complex task of connecting the 3 fields in an atomic tuple (0.46 F1 score, 0.59 precision, 0.44 recall). We defend that—for such a composite task—it is more important to have higher precision (less wrong predicted annotations) at the expense of recall (missing some existing annotations). The model produces few results, but in general, they are of good quality. Performances can improve by augmenting the training dataset; this indeed occurs thanks to the use of the CoVEffect Web Application presented later in the article.

## Results

Results include a double contribution: on the one hand, we provide complete predictions on a set of more than 7,000 abstracts from CORD-19 that are relevant to SARS-CoV-2 variation effects; on the other hand, we provide a user-friendly framework for expert users to annotate abstracts of interest and possibly contribute to additional training of the learning model.

### Annotation of the biology-related CORD-19 cluster

Abstracts informing about SARS-CoV-2 variation effects can be selected from CORD-19 via a 2-step process: (i) identification of a biology-related cluster and (ii) targeted search on the cluster based on particular keywords.

*Clusters*. We built a clustering model to partition in topic-based classes the CORD-19 dataset curated by our provisioning pipeline. For this purpose, we exploited the SPECTER document-level embeddings dataset distributed as part of CORD-19 (previously described in the *similarity-based discovery*). Because of the considerable size of the dataset, we opted for a representative-based clustering model (i.e., K-means). SPECTER embedding vectors are known to be effective in predicting the topic class associated with a paper [52]. Differently from [52], we did not know *a priori* the number of topic classes to be predicted. To choose an appropriate value for the number of clusters *k* of K-means, we plotted the silhouette score and the distortion for each candidate number of clusters, ranging from 2 to 50. The value K = 5 was chosen as it allowed us to visualize a spike in the plot of the silhouette score and an elbow-like shape in the plot of the distortion. For each of the 5 clusters, we generated *WordCloud* plots, including the most frequent words in papers’ titles abstracts and titles (top words common to clusters were excluded). This allowed us to manually recognize a 100 K abstracts cluster as the one mostly related to biological aspects.

*Keywords*. Out of the biology-related subset of CORD-19, we only targeted abstracts whose content relates to mutation and variants effects—the focus of CoVEffect. To this end, we described the subset of interest with a logical query expressed through the *Whoosh* search library [54]—previously mentioned for the *keyword-based search* of the data provisioning pipeline. The library already includes simple lemmatization capabilities; additionally, we loaded the OperatorsPlugin (which adds logical operators such as AND, OR, NOT), the GroupPlugin (to group search clauses using parentheses), and the SingleQuotePlugin (to specify single terms containing spaces by enclosing them in single quotes). Finally, we added a union set operation for the papers retrieved with each single query (equivalent to having all the queries in OR but without overloading the parsing process of *Whoosh*).

As a result of this procedure—employing the keyword-based query listed in the AdditionalFile7-keywords_query_list [63]—we could extract 7,230 papers from the cluster on biological aspects (see AdditionalFile8-CORD-19_batch_dataset_metadata [63]). We then ran the CoVEffect prediction on this dataset; the resulting predictions for the 7,230 abstracts are provided in AdditionalFile9-CORD-19_batch_dataset_prediction [63] as a contribution to the scientific community.

#### Testing results

Out of this batch, we tested the prediction performances on 100 randomly selected papers, ensuring that they did not overlap with the previously used training and validation sets. For these, we manually prepared target annotations (see AdditionalFile10-test_dataset_target [63]). Then, we predicted the annotations of their abstracts using our model (see AdditionalFile11-test_dataset_prediction [63]).

In Table 3, we show the results on the 100 papers of the test dataset, based on the comparison between target and predicted annotations. Reassuringly, performances were comparable to the ones obtained on the validation set. Indeed, they were only worse in the case of *entities*, whereas *effects*, *levels*, and also whole tuples improved their scores.

**Table 3:** Test set results (run to evaluate the predictions on 100 abstracts randomly selected from the CORD-19 biology-related cluster)

Task	Measure	F1 score	Precision	Recall
**Entity**	Average Weighted average	0.762 0.688	0.802 0.822	0.755 0.822
**Effect**	Average Weighted average	0.792 0.656	0.855 0.656	0.781 0.656
**Level**	Average Weighted average	0.832 0.624	0.832 0.625	0.832 0.624
**Whole tuple**	Average Weighted average	0.578 0.324	0.631 0.440	0.569 0.288

#### Benchmarking considerations

As mentioned in the “Related Work” section, Singhal et al. [32] previously proposed a method for extracting entities and relationships from biomedical text; that approach is considered today’s state-of-the-art. We do not compare our results with that approach because CoVEffect performs a significantly different task, providing an output that could be read as the result of 4 separate steps: entity recognition (for mutations and variants), entity linking (protein with mutation), classification (effects and levels), and relation extraction (among the previously extracted information). In essence, CoVEffect should not be considered the best possible method for performing each one of these tasks. Instead, it offers an all-in-one annotation platform that allows experts to insert annotations manually or to inspect, correct, and eventually accept predictions of specific triples entity–effect–level. The proposed approach can be interpreted as a combination of automated extraction and crowdsourcing, as initially proposed in [69].

### The CoVEffect web application

As a second output, we implemented the CoVEffect web application; its front end provides 2 main functionalities: (i) a search interface for finding papers of interest and (ii) an interactive interface to label abstracts with a semiautomated framework. The first functionality is based on a back-end retrieval module, which uses the methods described in the “Data provisioning” section (i.e., keyword-based search and similarity-based search of papers). The second functionality is fueled by a back-end extraction module, which uses the prediction model described in the “Language model and task design” section and implements a framework for semiautomated data labeling by users, as detailed in the following.

#### Semiautomated data labeling framework

**Figure 4:**
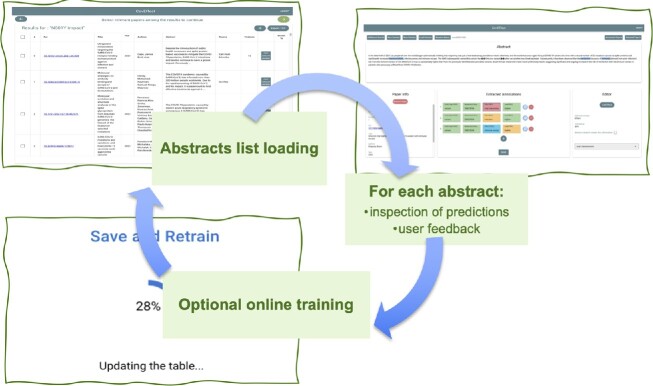
The iterative phases of the online semiautomated data labeling framework.

This framework aims to facilitate and accelerate the abstract annotation process operated by an expert researcher. A typical annotation session with iterative phases (shown in Fig. 4) follows.

- The user provides a list of abstracts.
- For each selected abstract: (i) the model generates a proposed labeling in the form of predicted tuples, and (ii) the user may edit each single prediction (i.e., 1 tuple field at a time).
- Once the editing session is over, the user is provided the choice of accepting the annotations and of retraining the model with the new provided annotations.

**Figure 5:**
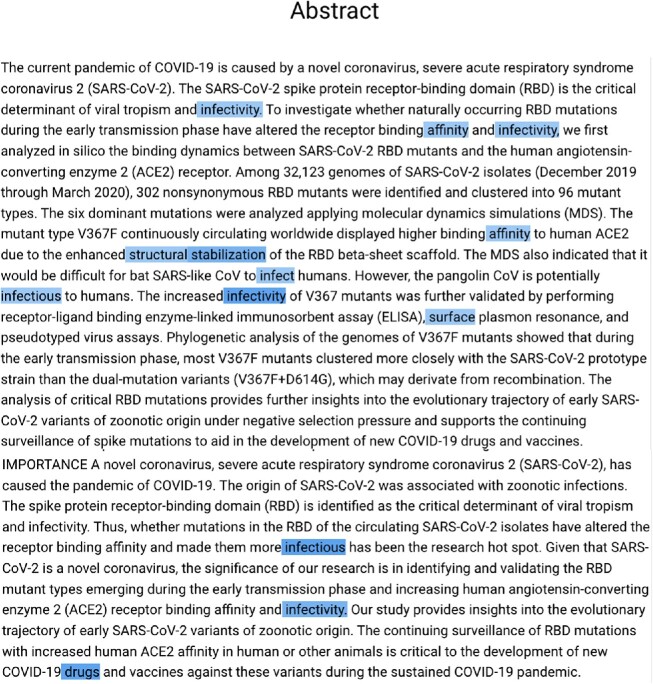
The gradient-based saliency map implemented in the CoVEffect tool. The example shows the abstract of the paper by Ou et al. [64] also used in Fig. 2 to motivate our task. The text fragments highlighted with different shades of blue are used by the model to predict the effect of the SPIKE_V367F mutation, here corresponding to the value “infectivity.”

The user may modify or add abstracts to the list at any point in time. For each prediction (type, entity, effect, or level), the framework provides 2 types of visual feedback. First, it shows the prediction confidence value with a color code: *green* for high confidence predictions >0.8, *yellow* for medium-confidence predictions between 0.6 and 0.8, and *black* for low-confidence predictions <0.6. Second, it shows a saliency map built on the input abstract. Saliency maps are a machine learning interpretation mechanism born in the field of explainable artificial intelligence; they are maps over the input that highlight the portions of the text that contributed the most to the extraction of given attributes. Here, we exploited the generation of saliency maps that employ the Gradient technique [70]. Such an idea was already proposed successfully in our previous work [45] where such a mechanism was well evaluated by the users of the system, as it allow users to understand whether a given result is not only predicted correctly, but also predicted by exploiting a correct information. As an example, in Fig. 5, we show the saliency map obtained for the prediction of the “infectivity” effect on the abstract of Ou et al. [64] previously introduced in Fig. 2.

#### Application workflow and example

**Figure 6:**
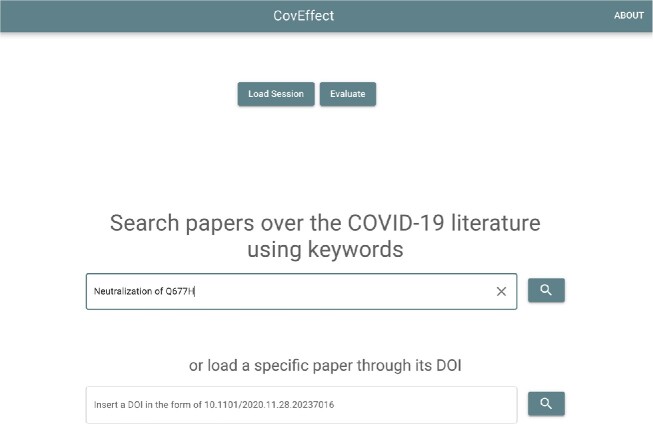
Homepage, with a section for keyword search and a section for DOI search.

**Figure 7:**
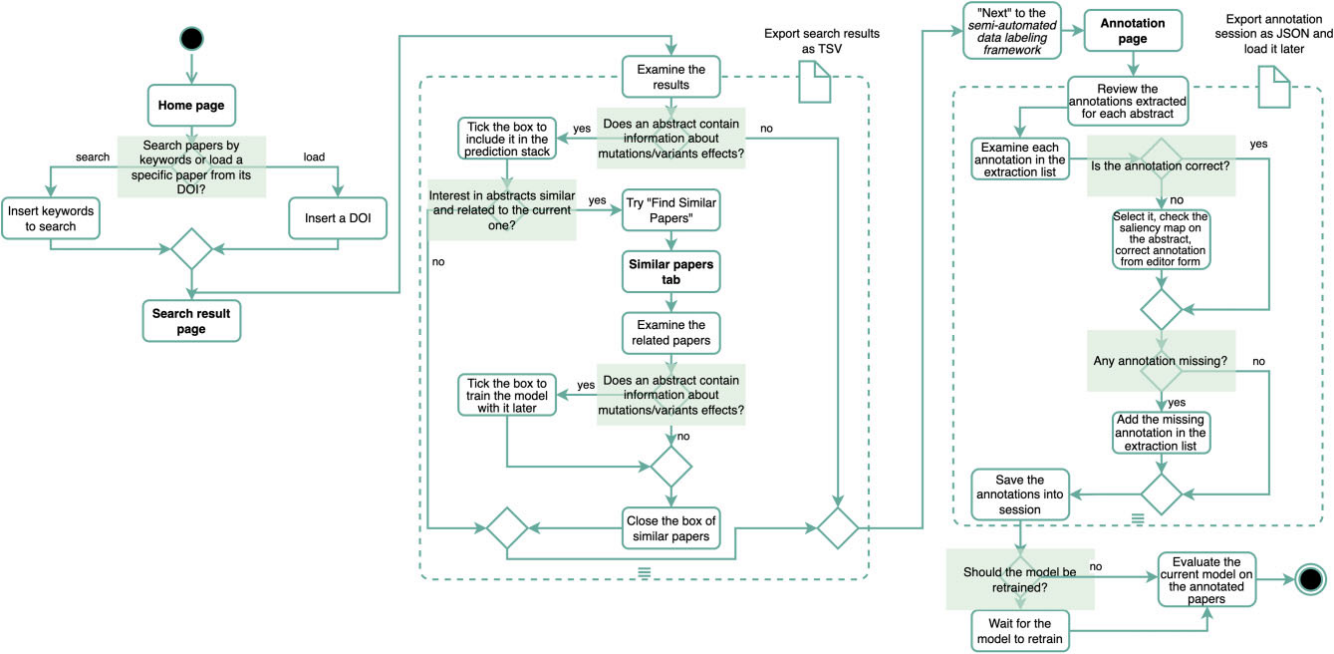
Activity diagram of the user’s interactions with the CoVEffect web application.

The “Homepage” of CoVEffect accepts 2 kinds of input: a list of keywords or a single DOI. Suppose that we search for the keywords “Neutralization of Q677H” (as shown in Fig. 6). The following workflow is explained by the activity diagram in Fig. 7.

**Figure 8:**
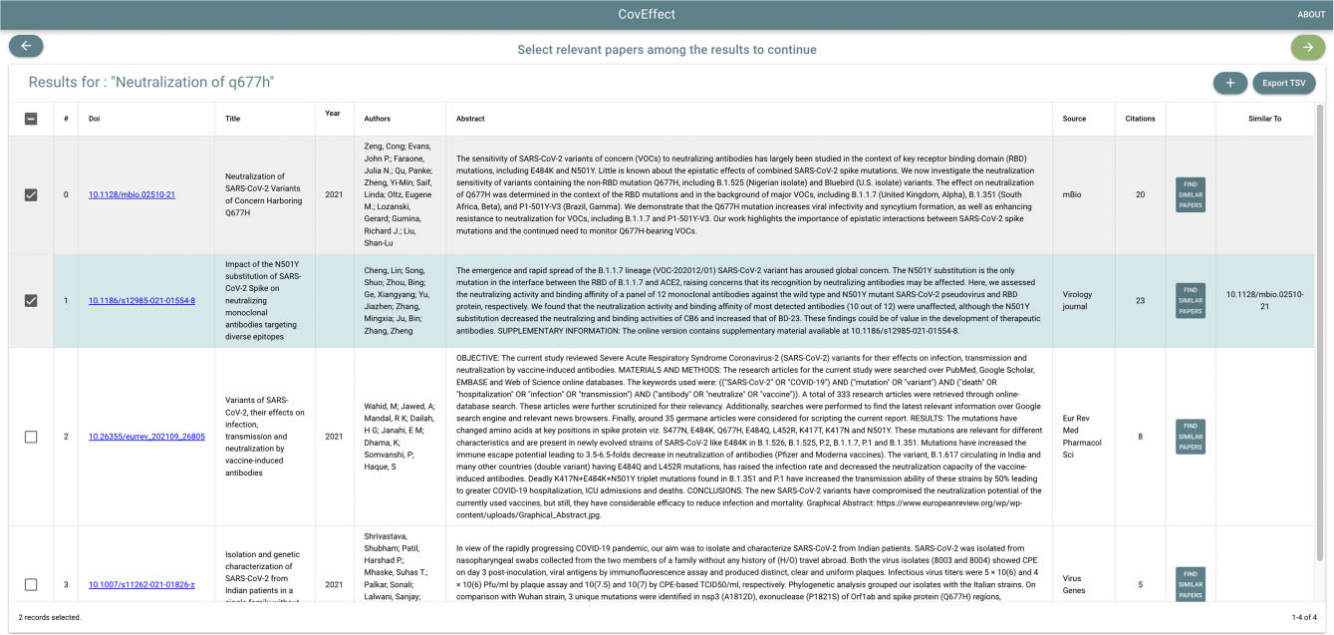
Paper List screen, obtained after searching for “Neutralization of Q677H” and inspecting papers similar to the first one (DOI “10.1128/mbio.02510–21” [71]). Papers that are selected by the user are highlighted in color: gray for the ones corresponding to the initial search, green for the ones corresponding to the similarity-based search.

Once the search is performed, we reach the “Search result page,” whose results can be examined (based on their metadata and abstract) and exported as a tab-separated file. Extracted papers may be of interest for the user (especially when they are focused on mutations or variants effects), in which case they can be included in the prediction stack. For each paper, users may also explore similar papers by opening the “Similar papers tab”; as before, papers of interest can be selected. When the user closes the tab, they will have a complete list of the searched papers, where papers selected are marked in gray and papers added for the similar ones are marked in green. Figure 8 shows an example where, from the papers obtained in the previous search, we selected the paper with DOI “10.1128/mbio.02510–21” [71] and its similar paper with DOI “10.1186/s12985-021-01,554–8” [72].

By pressing the green arrow on the top-right corner of the screen, we reach the “Annotation page.” This page allows users to inspect results and suggest changes for one abstract at a time. For each abstract, the framework extracts a list of predicted tuples, each composed of 4 fields (type, entity, effect, and level). For each of such annotations, the user can inspect the saliency map and decide if the annotation is correct (thus should be approved) or needs correction. Missing annotations can also be added manually.

**Figure 9:**
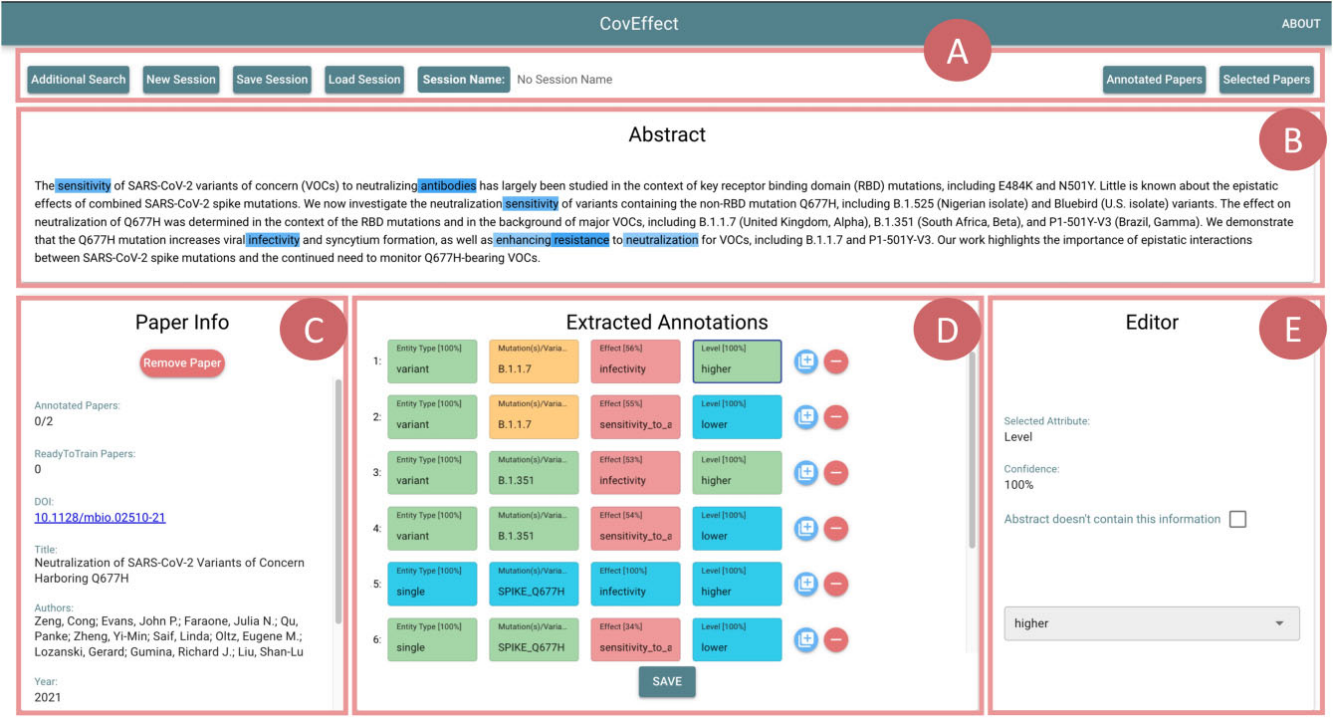
Overview of the CoVEffect interface, with a top bar and 4 panels, captured during the annotation of a paper by Zeng et al. [71]. Panel A includes the top bar; the commands on the left allow to return to the keyword search screen, open a new user session, save the current one, or load a previously closed one. The commands on the right allow to inspect the list of already processed papers or the list of papers selected through the keyword search. Panel B shows the abstract of the selected paper to be annotated, interactively highlighted using the gradient-based saliency map related to the tuple fragment selected in panel D. Panel C shows the metadata of the selected paper and the size of the stack of papers chosen by the user. Panel D shows the predicted tuples for the selected abstract, using the color-code for informing on the accuracy of the prediction. Panel E allows users to actively modify the prediction of the model and save the suggestions.

Figure 9 represents the status of the “Annotation page” for paper [71]. Panel A provides user utilities. Panel B shows the saliency map referring to the prediction of the value “higher” for the level of the first predicted tuple (selected in panel D). Panel C shows the metadata of the currently inspected paper and informs that the prediction stack contains 2 papers (of which none has yet been annotated, as we have not clicked on “SAVE”). Panel D shows predictions 1, 2, 3, 4, and 6 as produced by the prediction framework, with the exception of the level values of 2, 4, and 6 that have been manually corrected into the “lower” value (which had been wrongly predicted), by employing the drop-down menu in panel E.

In addition, a full tuple annotation has been added (number 5) regarding the single mutation Spike Q677H, which leads to an increase in infectivity of the SARS-CoV-2 virus.

When the user is satisfied with all the annotations associated with an abstract, these can be saved and are accordingly stored in the “Annotated Papers” list (panel A, top-right corner), where they can also be downloaded for further processing. Note that annotated abstracts that can be saved are the result of either a model prediction or of a user manual correction/addition.

When saving annotations for the first time, the user is prompted to name the current session. Sessions can be downloaded as JSON files and reloaded at a later time. Then, the user is asked if they wish to retrain the model immediately. This process is computationally intensive and may require several minutes based on the occupation of the servers. Users may also wait to annotate additional papers and retrain the model only at a later stage. The application can be installed on other machines using the Docker distribution available on our GitHub repository.

## Discussion

In this article, we described two contributions. On one hand, we provide the identification of SARS-CoV-2 variants and mutations’ effects over a relevant set of CORD-19 abstracts. On the other hand, we make this annotation extendable, as training data can be augmented by using the CoVEffect interface. The project stems from the need of providing a complete framework that supports semiautomatic extraction of structured information on SARS-CoV-2 variation effects. We had previously employed transformer-based text extraction for capturing key-value pairs from genomic experiments (from Gene Expression Omnibus). The task performed in this case is more complex, as it aims to identify attributes that are interdependent: mutation or variants with their effect and level.

A considerable improvement of the initial GPT2 model was necessary to address this new challenge. In addition, no preexisting training dataset was available; we thus designed a methodology to build a small manually crafted dataset of good quality. The trajectory to evaluate the performances of our method is as follows: we chose an initial dataset with minimal size, and at each small delta increase, we evaluated the changes in performances on a test dataset until a satisfactory result was reached. This process was necessary to find a trade-off between 2 needs: the minimization of the effort of expert manual annotation and maximization of prediction performances. This effort has paid off in terms of recognizing single concepts; however, the linked tuple prediction still has much room for improvement.

To inspect the most challenging aspects of the prediction task, we performed an error analysis divided into 3 categories: (i) entity name prediction (nonconstrained to any value, filtered with a RegEx filter), (ii) effect/level prediction (restricted to our taxonomy values), and (iii) association between entity, effect, and level. Table 4 presents an overview of the most representative errors each with an associated example.

**Table 4:** Typical issues detected in the prediction task. The first column groups issues by macro-category, the second describes the scenario that leads to an *Issue*, and the third and fourth provide the reference DOI to an abstract and a short text excerpt from the abstract. Words in orange show the relevant information for the expected values (Target) as opposed to the obtained prediction.

	Issue	DOI	Text excerpt from abstract	Target	Prediction
Entity name prediction	Uncommon naming (mutations/variants)	[73]	The S:655Y substitution was transmitted more efficiently than …	SPIKE_H655Y	—
	Mutations/variants referred to as a group	[71]	… major VOCs, including Alpha, Beta, and Gamma. We demonstrate that the Q677H mutation increases viral infectivity and syncytium formation, as well as enhancing resistance to neutralization for VOCs.	Alpha	Alpha
				Beta	Beta
				Gamma	—
	Mutations/variants reported as long lists	[74]	To understand the impact of spike protein mutations on the binding interactions required for virus infection and the effectiveness of neutralizing monoclonal antibody (mAb) therapies, mutants D614G, N501Y, N439K, Y453F, and E484K were assessed.	SPIKE_D614G	—
				SPIKE_N501Y	SPIKE_N501Y
				SPIKE_N439K	—
				SPIKE_Y453F	SPIKE_Y453F
				SPIKE_E484K	SPIKE_E484K
Effect and/or level prediction	Effect terminology	[75]	The increased ACE2-binding affinity of variants containing the N501Y or E484K mutations can be traced to the time-dependent disruption and/or formation of interfacial salt bridges, not necessarily apparent from structural models but detected by extensive molecular dynamics simulations.	SPIKE_N501Y binding to host receptor	SPIKE_N501Y binding to host receptor
				SPIKE_N501Y intermolecular interactions	SPIKE_N501Y protein conformational optimization.
				SPIKE_E484K binding to host receptor	SPIKE_E484K binding to host receptor
				SPIKE_E484K intermolecular interactions	SPIKE_E484 protein conformational optimization.
	Vague results presentation	[73]	We demonstrate that the substitution S:655Y, represented in the Gamma and Omicron VOCs, enhances viral replication and spike protein cleavage. All VOCs tested exhibited increased spike cleavage and fusogenic capacity.	GAMMA viral replication	GAMMA viral replication
				GAMMA protein functioning	-
Association	Multiple effects connected to same entity	[76]	Infections caused by the delta variant increases the risk of hospitalization within 14 days after symptom onset, and the high viral load correlates with COVID-19 associated morbidity and mortality.	DELTA, risk of hospitalization	DELTA, risk of hospitalization
				DELTA, viral load	DELTA, viral load
				DELTA, fatality rate	-
	Different levels for same entity effect (not supported)	[77]	Naturally occurring variants in Orf3a (Q57H) and nsp2 (T85I) were associated with poor replication in Vero-CCL81 cells but not in BEpCs.	ORF3a_Q57H viral replication, lower	-
				ORF3a_Q57H viral replication, unaffected	ORF3a_Q57H viral replication, unaffected

Types of errors captured in the *entity name prediction* mainly occurred when the abstract included:

- *Mutation/variant named with uncommon terminology*. The typical way to name a mutation is to declare the protein where the mutation occurred followed by a mutation signature (⟨reference amino acid, coordinate in protein, alternative amino acid⟩, e.g., Spike D614G). The most adopted terminologies to name a SARS-CoV-2 variant are Pango lineages [61] or WHO Greek letters [20]; however, there are other ways to refer to variants (e.g., GISAID or Nextstrain clades), which are currently not supported in CoVEffect. Table 4 shows an example from [73] where a different naming scheme is used for a mutation of interest, which makes the model’s mission harder.
- *Effect/level associated with a named group of variants*. The WHO has classified variants into variants of concern and other classes according to their impacts [20]. In publications, we often find reference to effects studied on a group of variants, referred to with such terms. Table 4 shows one such case [71], where CoVEffect can miss 1 or more entities in the list.
- *Mutations/variants written as long lists*. Some publications—noticeably the ones using computational methods to analyze their variants of interest—tend to deal with long lists of mutations. CoVEffect model may miss some entities in such scenarios (as happened in [74]).

Moreover, issues occurring in the *entity/level prediction* mainly occurred when the abstract included:

- *Effects misclassification*. The model does not always recognize effects as they are expressed in our taxonomy, especially when there exist connections between different effects. This case may happen when an effect is a special case of another effect (e.g., binding to a host receptor is a special case of a host–virus interaction); in this case, only using a broad context and expert user knowledge does it become possible to understand the correct target effect. Table 4 shows 1 such example from [75].
- *Levels misclassification*. The changes of some effects are more easily expressible through the higher/lower comparators (i.e., higher transmissibility, lower severity). Unfortunately for other effects (e.g., protein conformational optimization), comparators are less used in text.
- *Unclear results presentations*. Effects reported in abstracts with a vague presentation of the results can be missed. For example, some publications that report on the effectiveness of a specific therapeutic measure might not declare that the measure is indeed a drug. Other publications (see [73] for an example) study the effect of a mutation on the functions of viral proteins without making explicit that the topic discussed is a protein function—making it hard for the model to predict the effect.

Finally, problems occurring in predicting the *association between an entity and its effect level* mainly occurred when the abstract included:

- *Multiple effects for 1 entity*. The model can miss the association of 1 (or more) effects that are part of a list (as happened in [76]).
- *Multiple levels for 1 entity effect*. Given abstracts may include the specification of an entity and associated effect with multiple levels (e.g., in [77]). This scenario is likely to be found when the specific effect has been studied under multiple conditions (e.g., measuring the viral loads of a variant in different tissues or studying the binding of a specific variant with a wide range of antibodies). CoVEffect current data model does not support multiple disagreeing levels for an entity–effect pair. This impacts on the recall of our results.

Notably, the prediction model reached quite good performances, as shown in Tables 2 and 3, and still has much space for improvement thanks to the expected enhancements on the training dataset. An interesting result is that mutation entities were very well predicted even when the protein information was far apart in the text from the mutation signature (see our motivating example in Fig. 2, where Spike is far from V367F, but they are correctly associated); the interpretability mechanism of saliency maps is of great support to highlight these cases. Moreover, the model worked well in detecting our targets: protein amino acid–based mutations rather than genomic nucleotide-based mutations and lineages rather than clades.

CoVEffect brings a number of tangible results to the scientific community, which we here describe. Immediate integrated use of our resulting annotated database was made within our CoV2K [22] system by updating the AA_changes, Variant, and Effect entities. Other data-driven analysis resources developed by our group (such as VirusViz [78] and ViruClust [79]) could immediately benefit from the addition of structured tuples connecting mutations and effects. At the same time, any other resource employed in the current practice of virologists and phylogenetists (such as CoVSpectrum [80] and Outbreak.info [81]), studying the trend of specific mutations and variants, can benefit from the provisioning of a dataset with this structured information. Our output can be appreciated in the AdditionalFile9 [63], containing the predicted annotations for the whole biology-related CORD-19 cluster. External users may also annotate other abstracts by installing CoVEffect through our Docker distribution and running the batch annotator (available as a Python notebook on our GitHub repository).

Next, we aim to extend the scope of CoVEffect by including the possibility of recognizing also alternative formulations of mutation and variant names, tuples reporting on different levels for the same entity and effect, groups of mutations leading collaboratively to the same effect, insertions and deletions, the method used to establish the effect (epidemiological, experimental, computational or inferred), and effects reported with complex—possibly quantitative—formulations. We will also add a “mutation validation” module to check the semantic consistency of mutation signatures, on top of the RegEx-based check.

In the future, we aim to apply CoVEffect to other subparts of the CORD-19 dataset as well as to expand to other literature corpuses, focusing on different, well-defined, and delimited domains. More in general, our framework is suitable to resolve similar problems where the prediction task attempts to recognize in text the associations between given entities and related values (within existing taxonomies). One additional possibility regards predicting tuples of individual mutations, with their associated genetic background, and their mutual interaction; this has been demonstrated to be important for SARS-CoV-2, possibly supporting the explanation/prediction of new variants.

## Availability of Source Code and Requirements

Project name: CoVEffectProject

Homepage: https://gmql.eu/coveffect/

Code repository: https://github.com/armando2603/coveffect/

Operating system: Platform independent

Programming language: The source code of the data provisioning module and the deep learning–based prediction framework are implemented in Python. The CoVEffect web interface to annotate abstracts is implemented in Python (Flask framework) and JavaScript (Vue framework).

Other requirements: The application can be installed on any machine with its Docker image version.

License: MIT

RRID:SCR_023415

biotools ID: CoVEffect

## Supplementary Material

giad036_GIGA-D-22-00331_Original_Submission

giad036_GIGA-D-22-00331_Revision_1

giad036_Response_to_Reviewer_Comments_Original_Submission

giad036_Reviewer_1_Report_Original_Submission Bahrad Sokhansanj -- 1/30/2023 Reviewed

giad036_Reviewer_2_Report_Original_Submission Theo Sanderson -- 2/14/2023 Reviewed

## Data Availability

All supporting data and materials are available in the *GigaScience* GigaDB database [63] and on Zenodo [82].

## Additional Files

**AdditionalFile1-effects-taxonomy**. Descriptions of legal values for the “Effect” field, based on a categorized taxonomy.

**AdditionalFile2-levels-taxonomy**. Descriptions of legal values for the “Level” field.

**AdditionalFile3-training_dataset_target**. List of target tuples (manually annotated) of 221 abstracts considered for training the model. For each abstract, target tuples follow the schema ID, DOI, title, entity, effect, level, type (mutation or variant), and tuples_count (>1 when an effect/level is shared by multiple entities, #abstracts containing the same effect described in the tuple).

**AdditionalFile4-validation_dataset_target**. List of target tuples (manually annotated) of 50 abstracts considered for validating the prepared prediction model. For each abstract, target tuples follow the schema defined for AdditionalFile3.

**AdditionalFile5-validation_dataset_highlighted**. Textual abstracts of the 50 manuscripts considered for validation; the text used to support the manual target annotations has been highlighted in yellow.

**AdditionalFile6-validation_dataset_prediction**. List of predicted annotations of 50 abstracts considered for validating the prepared prediction model; it contains 4 sheets, respectively for entity, effect, level, and whole tuple predictions.

**AdditionalFile7-keywords_query_list**. Keyword-based search run on the CORD-19 dataset to extract a relevant subset of abstracts regarding the scope of interest of CoVEffect. The Boolean logic used to combine keywords is explained in the section “Annotations of the biology-related CORD-19 cluster.”

**AdditionalFile8-CORD-19_batch_dataset_metadata**. Metadata of the 7,230 papers extracted by the keyword-based query in AdditionalFile7. These abstracts have been annotated by the prediction framework.

**AdditionalFile9-CORD-19_batch_dataset_prediction**. List of predicted annotations of 7,230 abstracts extracted from the biology-related cluster of CORD-19.

**AdditionalFile10-test_dataset_target**. List of target tuples (manually annotated) of 100 abstracts randomly selected from the 7,230 extracted as in AdditionalFile8. For each abstract, target tuples follow the schema defined for AdditionalFile3.

**AdditionalFile11-test_dataset_prediction**. List of predicted annotations of 100 abstracts considered for testing the prediction model on a subset of the CORD-19 biology-related cluster. As AdditionalFile6, it contains 4 sheets, respectively for entity, effect, level, and whole tuple predictions.

## Abbreviations

CDC: Centers for Disease Control and Prevention; CORD-19: COVID-19 Open Research Dataset; GPT2: Generative Pre-trained Transformer 2; NER: Named Entity Recognition; NLP: Natural Language Processing; WHO: World Health Organization.
