# Supplementary material for: CoVEffect: interactive system for mining the effects of SARS-CoV-2 mutations and variants based on deep learning
Source: Gigascience. 2023 May 23;12:giad036. doi: 10.1093/gigascience/giad036 (PMC10205000; doi:10.1093/gigascience/giad036)
Supplement: giad036_GIGA-D-22-00331_Original_Submission [file giad036_giga-d-22-00331_original_submission.pdf]

GigaScience

# CoVEffect: Interactive System for Mining the Effects of SARS-CoV-2 Mutations and Variants Based on Deep Learning

--Manuscript Draft--

| | |
|---|---|
| Manuscript Number: | GIGA-D-22-00331 |
| Full Title: | CoVEffect: Interactive System for Mining the Effects of SARS-CoV-2 Mutations and Variants Based on Deep Learning |
| Article Type: | Research |
| Funding Information: | Horizon 2020 Framework Programme (693174) — Prof Stefano Ceri |
| Abstract: | Background: Literature about SARS-CoV-2 widely discusses the effects of variations that spread in the last three years. Such information is dispersed in the texts of several research articles, hindering the possibility of practically integrating it with related datasets (e.g., millions of SARS-CoV-2 sequences available to the community). We aim to fill this gap, by mining literature abstracts to extract -- for each variant/mutation -- its related effects (in epidemiological, immunological, clinical, or viral kinetics terms) with labeled higher/lower levels in relation to the non-mutated virus.<br>Results: The proposed framework comprises: i) the provisioning of abstracts from a COVID-19-related big data corpus (CORD-19), and ii) the identification of mutation/variant effects in abstracts using a GPT2-based prediction model. The above techniques enable the prediction of mutations/variants with their effects and levels in two distinct scenarios: (a) the batch annotation of the most relevant CORD-19 abstracts, and (b) the on-demand annotation of any user-selected CORD-19 abstract through the CoVEffect Web application (http://gmql.eu/coveffect/), which assists expert users with semi-automated data labeling. On the interface, users can inspect the predictions and correct them;<br>user inputs can then extend the training dataset used by the prediction model. Our prototype model was trained through a carefully designed process, using a minimal and highly diversified pool of samples.<br>Conclusions: The CoVEffect interface serves for the assisted annotation of abstracts, allowing the download of curated datasets for further use in data integration or analysis pipelines. The overall framework can be adapted to resolve similar unstructured-to-structured text translation tasks, which are typical of biomedical domains. |
| Corresponding Author: | Anna Bernasconi, Ph.D.<br>Politecnico di Milano<br>Milano, Lombardia ITALY |
| Corresponding Author Secondary Information: | |
| Corresponding Author's Institution: | Politecnico di Milano |
| Corresponding Author's Secondary Institution: | |
| First Author: | Giuseppe Serna García |
| First Author Secondary Information: | |
| Order of Authors: | Giuseppe Serna García |
| | Ruba Al Khalaf |
| | Francesco Invernici |
| | Stefano Ceri, Ph.D. |
| | Anna Bernasconi, Ph.D. |
| Order of Authors Secondary Information: | |
| Additional Information: | |

Powered by Editorial Manager® and ProduXion Manager® from Aries Systems Corporation

| Question | Response |
|---|---|
| Are you submitting this manuscript to a special series or article collection? | No |
| **Experimental design and statistics**<br><br>Full details of the experimental design and statistical methods used should be given in the Methods section, as detailed in our Minimum Standards Reporting Checklist. Information essential to interpreting the data presented should be made available in the figure legends.<br><br>Have you included all the information requested in your manuscript? | Yes |
| **Resources**<br><br>A description of all resources used, including antibodies, cell lines, animals and software tools, with enough information to allow them to be uniquely identified, should be included in the Methods section. Authors are strongly encouraged to cite Research Resource Identifiers (RRIDs) for antibodies, model organisms and tools, where possible.<br><br>Have you included the information requested as detailed in our Minimum Standards Reporting Checklist? | Yes |
| **Availability of data and materials**<br><br>All datasets and code on which the conclusions of the paper rely must be either included in your submission or deposited in publicly available repositories (where available and ethically appropriate), referencing such data using a unique identifier in the references and in the "Availability of Data and Materials" section of your manuscript. | Yes |

| | |
|---|---|
| Have you have met the above requirement as detailed in our Minimum Standards Reporting Checklist? | |

Manuscript Click here to access/download;Manuscript;manuscript.pdf

Placeholder for OUP logo oup.pdf

Placeholder for journal logo gigascience-logo.pdf

*GigaScience*, 2022, 1–14

doi: xx.xxxx/xxxx
**Manuscript in Preparation**
**Research**

RESEARCH

# CoVEffect: Interactive System for Mining the Effects of SARS-CoV-2 Mutations and Variants Based on Deep Learning

Giuseppe Serna García[1], Ruba Al Khalaf[1], Francesco Invernici[1], Stefano Ceri[1] and Anna Bernasconi[1,*]

[1]Dipartimento di Elettronica, Informazione e Bioingegneria – Politecnico di Milano

[*]anna.bernasconi@polimi.it

## Abstract

**Background:** Literature about SARS-CoV-2 widely discusses the effects of variations that spread in the last three years. Such information is dispersed in the texts of several research articles, hindering the possibility of practically integrating it with related datasets (e.g., millions of SARS-CoV-2 sequences available to the community). We aim to fill this gap, by mining literature abstracts to extract – for each variant/mutation – its related effects (in epidemiological, immunological, clinical, or viral kinetics terms) with labeled higher/lower levels in relation to the non-mutated virus. **Results:** The proposed framework comprises: i) the provisioning of abstracts from a COVID-19-related big data corpus (CORD-19), and ii) the identification of mutation/variant effects in abstracts using a GPT2-based prediction model. The above techniques enable the prediction of mutations/variants with their effects and levels in two distinct scenarios: (a) the batch annotation of the most relevant CORD-19 abstracts, and (b) the on-demand annotation of any user-selected CORD-19 abstract through the CoVEffect Web application (http://gmql.eu/coveffect/), which assists expert users with semi-automated data labeling. On the interface, users can inspect the predictions and correct them; user inputs can then extend the training dataset used by the prediction model. Our prototype model was trained through a carefully designed process, using a minimal and highly diversified pool of samples. **Conclusions:** The CoVEffect interface serves for the assisted annotation of abstracts, allowing the download of curated datasets for further use in data integration or analysis pipelines. The overall framework can be adapted to resolve similar unstructured-to-structured text translation tasks, which are typical of biomedical domains.

**Key words**: Deep Learning; Language Models; Machine Learning Interpretability; CORD-19 Dataset; SARS-CoV-2; Viral Variants; Viral Mutations; Web Interface

## Introduction

The COVID-19 pandemic has brought unprecedented attention of the research community towards the mechanisms of the virus, including its variation, spread, and impacts on the host immune system. At the start of 2020, it was estimated that 200,000 coronavirus-related journal articles and preprints would be published by the end of the year [1]. As of today, about 3 years since the beginning of the pandemic, more than one million articles have become available.

This wide COVID-19 related literature is still largely unexplored, but can be employed for data and text analysis. Most COVID-19 research outputs have been gathered within the COVID-19 Open Research Dataset (CORD-19 [2]) by the Allen Institute. The corpus includes preprints and papers from Semantic Scholar up to mid-2022, sourced from PubMedCentral, PubMed, the World Health Organization's Covid-19 Database, and the preprint servers bioRxiv, medRxiv, and arXiv.

In parallel, there has been a worldwide spread of open data representing SARS-CoV-2 sequences (through the data sources GISAID [3], GenBank [4] and COG-UK [5]), gathered on repositories

**Compiled on:** December 5, 2022.
Draft manuscript prepared by the author.

by public and private institutes. The study of viral sequences has allowed to target several research questions related to the epidemiology and immunology aspects of the viral spread [6, 7, 8]. Much attention has also been dedicated to identifying amino acid-level mutations (or groups of them – coordinated within variants) that lead to particular changes in the behavior of the virus and its ability to establish infections – when compared to the wild type [9, 10, 11]. Note that, currently, it is hard to integrate data about sequences (with associated mutations) with information about variation effects, as the latter is not available in structured formats.

Structured information can be retrieved resorting to Natural Language Processing (NLP) techniques. NLP models usually require a considerable quantity of training data to learn their tasks. However, recent breakthroughs with deep learning models such as the Generative Pretrained Transformer (e.g GPT2 [12]) allowed to build multi-task learners that use fewer data than classic supervised machine learning techniques.

In this work, we use GPT2 to learn tuples that contain a SARS-CoV-2 variation, its effect and level, starting from CORD-19 abstracts. The model is trained on a small dataset that we carefully fabricated, as no such ready-to-use dataset was available. As our system enables expert users to provide more input annotations, it is preferable to use a model that dynamically and efficiently learns how to handle new annotations over time; in parallel, it is desirable to augment the training dataset in a continuous manner. To allow for this, we use a semi-automated data labeling system, which employs the predictive model to assist the human labeller, combining manual annotations with automatic tuples extraction. The model is used to recommend labels and automate basic functions in a labeling interface. The user can decide when to employ the generated labeled data for autmenting the training dataset and re-training the model. A user-friendly web interface (http://gmql.eu/coveffect/) allows expert users to annotate abstracts with variation effects without requiring any programming or data management knowledge.

## Related Work

Currently, the task of recognizing mutations and variants' effects needs to be performed by hand. There are a very few resources that provide this kind of information; when this is the case, they are exclusively manually curated. FaviCoV and ESC [13, 14] respectively store SARS-CoV-2 genetic mutations that are functionally relevant and are associated with immune escape. The antigenic role of amino acid replacements in the context of the human immune response is also the focus of the COG-UK Mutation Explorer [15], while a list from the WHO concentrates on specific replacements that characterize variants [16]. Torrens-Fontanals et al. report on how variation impacts can be predicted [17]. Online resources such as CoVariants [18], ECDC [19], WHO [20], and CDC [21] explain variants' effects commenting how they are reported in the literature. We previously made extensive curation of effects stored in CoV2K [22], a knowledge base of data and knowledge about SARS-CoV-2; our cumbersome manual curation approach has quickly become unfeasible, prompting us to explore alternative solutions.

Several NLP techniques have been used and adapted to bioinformatics-relevant problems [23], with few studies addressing results' explainability [24]. Less approaches have employed transformer-based techniques for biomedical text extraction tasks [25], mainly focusing on connections between entities [26, 27].

In our past work [28, 29], we employed deep learning transformer-based techniques for NLP to infer attributes from Gene Expression Omnibus [30] experiment metadata, formulating the problem as a translation task. Cannizzaro et al. [28] and Serna et al. [29] achieved the result of translating Gene Expression Omnibus experiment descriptions into key:value pairs (e.g., cell line:K562, disease:myeloid leukemia, assembly:hg19, assay:Chip-Seq, target: H3K9me3).

CoVEffect stems from this thread of works, but it is carefully adapted to solve a more complex task, i.e., the one of predicting a series of tuples from SARS-CoV-2-related abstracts where we consider a variation, its effect, and the change of its level. Currently available systems are either only supporting user-driven annotation [31], predictions of single independent annotations with ontological terms [32], or biomedical general-purpose triplets based on existing knowledge graphs [33] especially targeted to protein-protein interactions [34]. These correspond to different tasks than the one performed by CoVEffect and the described approaches do not allow for online modifications of the training dataset. Our purpose is closer in spirit to the one targeted in Mahajan et al. [35]; however their work is focused on clinical aspects (text is extracted from Electronic Health Records instead of research abstracts) and is not supported by a user-oriented interface.

All in all, to the best of our knowledge, CoVEffect is one of the first transformer-based approaches applied to biomedical tasks, combined with explainability approaches.

## Materials and Methods

Figure 1 captures the high-level architecture of the whole framework. As our input, we consider the wealth of information contained in the CORD-19 dataset. From the data corpus we only extract abstracts that reach sufficient quality standards and provide essential metadata.

Two offline processes exploit the dataset: 1) *data provisioning*, where we perform data curation and prepare a dataset that supports indexed keyword-based search and similarity-based search; 2) *prediction model setup*, where we manually craft a dataset, use it for training the model, check its performances (through a validation dataset) and evaluate the need to change or augment the initial training dataset.

The artifacts produced by these two processes are the indexed curated dataset of CORD-19 abstracts and the trained prediction model. They feed two possible modes of use, sharing standardized output formats:

- an offline *Batch Annotator*, which provides annotated data for a selection of 7,230 relevant abstracts from the CORD-19 corpus;
- an interactive online *Web Application* employed by expert users to annotate samples and inspect predicted annotations.

### Data provisioning

From the latest and final CORD-19 release (issued in June 2022) we collected *metadata.csv*, a table with metadata of all papers, and *cord_19_embeddings.tar.gz*, a collection of pre-computed SPECTER [36] document embeddings for each paper. The data provisioning pipeline aims to produce a curated set of abstracts (equipped with metadata) to support the activities of the learning framework.

#### *Data curation*

As described by Wang et al. [2], the CORD-19 dataset gathers COVID-19-related papers from several sources. In this dataset, papers are already harmonized and de-duplicated: in the metadata table each *cord_uid* represents a cluster of papers with colliding identifiers, such as *DOI* or *arxiv_id*. For our system, we extracted a portion of the original CORD-19 dataset: we kept only one record for each paper, thereby avoiding duplicated entries and easing the annotation user experience. To this end, we designed a reconciliation step: for each cluster, we favored the entry with the longest abstract and promoted values from other members of the cluster to fill in the missing information; then, we removed the other members of the cluster, obtaining only one entry for each paper. We also removed

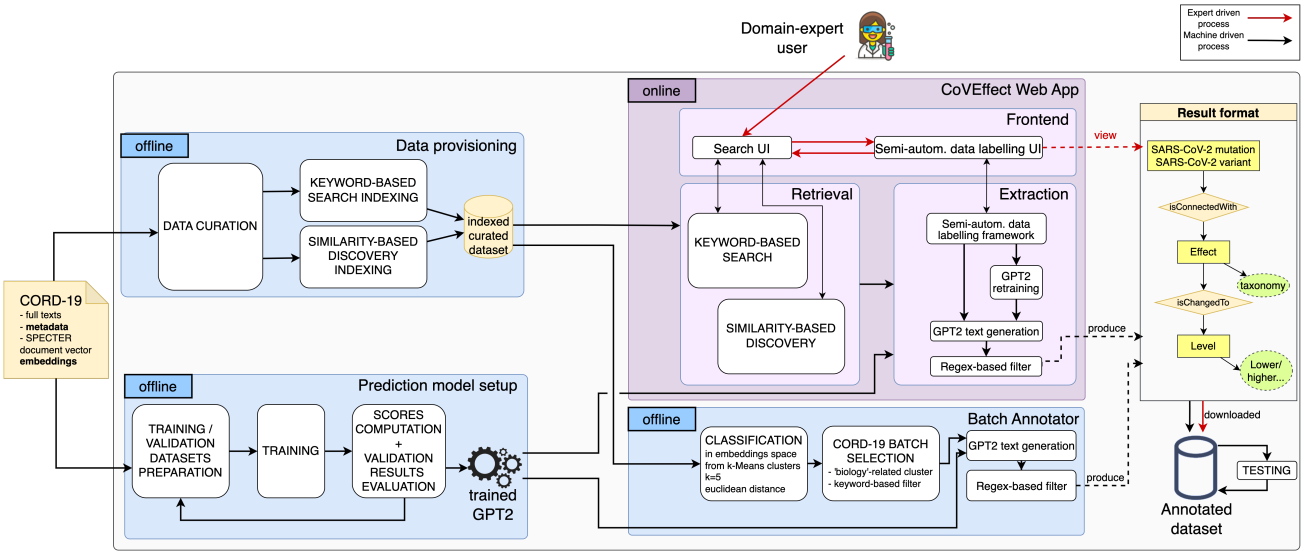


**Figure 1.** CoVEffect framework overview.

those papers for which an abstract was not available. Additionally, we used *langdetect* [37] – a language detection library ported from Google's *language-detection* – to detect the language of the abstracts and filtered out the papers not written in English.

### *Abstracts retrieval*

The curated dataset has been indexed to support search on the paper abstracts. Such step is functional to the retrieval task of the learning system, where the user searches abstracts that are of interest. For the purpose, we built a search engine leveraging two existing libraries.

- The *keyword-based search* is based on *Whoosh* [38], a full-text indexing and searching library, to let users search the abstracts using combinations of keywords.
- The *similarity-based discovery* is based on *Annoy* [39], an approximate nearest neighbor search library, to let the users discover abstracts similar to those already selected. These recommendations are computed by exploiting the SPECTER embeddings of the papers, which are document-level vector representations originated from citation-based transformers. For our purpose, we dramatically reduced the dimensionality of the vector space from 768 to 100. The 100 dimensions were selected by means of a Principal Component Analysis, resulting into a representation with explained variance ratio of 74%. In line with the recommendation task overviewed in [36], we chose cosine similarity as a similarity metric among papers, by setting the distance parameter of the AnnoyIndex to '`angular`'.

## Language model and task design

### *Model*

In this work, we favoured text-generative transformer models over BERT-like models [40] because of their ability to perform multi-task learning [12] and to easily adapt to new tasks. Indeed, text-generative models formulate multitask learning as a conditional distribution $P(output|input, task)$, where the task to be performed can be easily expressed in the form of text. We also make a distinction between general and domain-specific pretrained models. General models are usually pretrained with large datasets aiming to be as general as possible (e.g, BookCorpus [41] and English Wikipedia). Domain-specific models, instead, are further pretrained in order to fit a particular application (e.g., medicine, biology). In our case the specific domain knowledge is represented by the CORD-19 dataset [2]. In the last years, several new generative models have been proposed (e.g T5, BART, GTP3, BLOOM). These models achieved increasingly better performances, mostly by increasing the size of the model parameters and the size of the pretraining datasets. As a trade-off, bigger models are significantly slower.

In our work, the model is also used in an interactive way (with a domain expert), thus we preferred smaller models to large models. Considering all these aspects, we opted for a domain-specific version of a gpt2-small model available on the huggingface model hub [42]; it represents a reasonable compromise between model size and performances in a very specific domain. We propose it as a baseline for future works that could make use of our dataset.

### *Target data format*

Abstracts are annotated by recognizing structured tuples of the form $\langle type, entity, effect, level\rangle$. Possible *types* are "mutation" and "variant". With *mutation*, we refer to amino acid changes within specific proteins, occurring in a position where a reference residue has been changed into an alternative residue. These changes correspond to non-synonymous nucleotide mutations; we do not consider synonymous nucleotide mutations, as they typically do not influence the protein functionalities. In this work, we focus on substitutions, leaving aside insertions and deletions as they would require substantial additional training due to their very heterogeneous formulations. With *variant*, we denote forms of the SARS-CoV-2 that are considerably different from the original wild-type [43], as they accumulated a set of amino acid changes that characterize their phenotypic characteristics [44]. Variants are typically associated with a name to easily address them.

In our tuples, *entities* are the names of mutations (e.g., Spike_N501Y or NSP12_P323L) or of variants, e.g., Alpha, Delta, Omicron (as named by WHO [20]) or B.1.1.7, B.1.617, B.1.519 (as named by Pangolin [45]).

*Effects* are chosen from a taxonomy, i.e., a controlled vocabulary of terms including for example transmissibility, disease severity, resistance to antiviral drugs, or change in the protein kinetics (flexibility or stability properties). We previously proposed an initial version of this vocabulary [22, 46], which has now evolved into a complete list of effects organized by category (either 'epidemiology', 'immunology', 'viral kinetics and dynamics', or 'diagnosis, prevention, and treatments'). The full list can be found the AdditionalFile1-effects-taxonomy [47].

Finally, each effect has an associated *level*, i.e., higher, lower, unaffected, undefined, no evidence (see AdditionalFile2-levels-taxonomy [47] for detailed definitions).

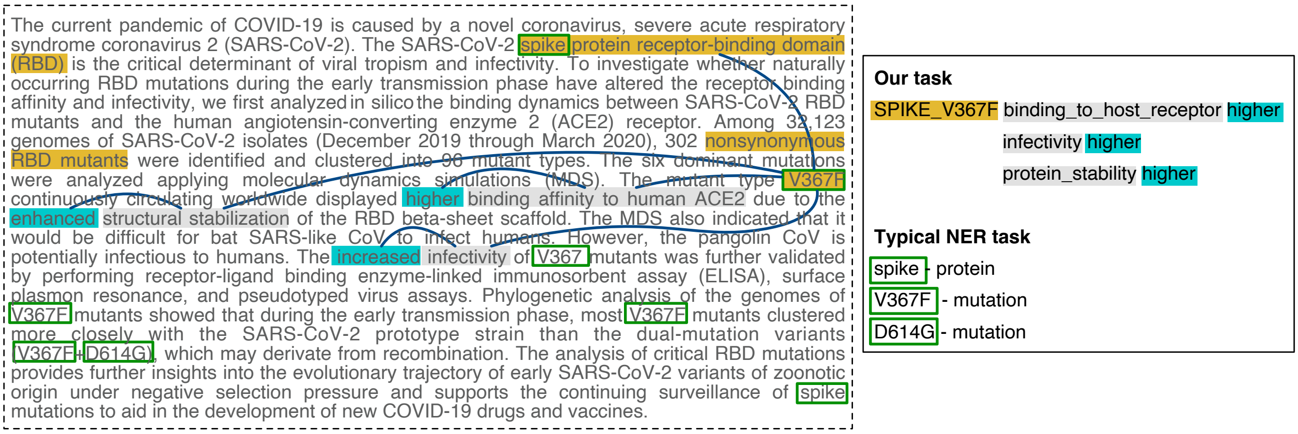


**Figure 2.** Difference between tasks resolved by a NER approach (only recognizing entities from a text excerpt) and our translation-based approach (targeting entities with connected effects and levels). The abstract excerpt is extracted by a paper on the *Journal of Virology* by Ou et al. [48], https://doi.org/10.1128/JVI.00617-21. Information used to form our tuples is connected through blue lines. Yellow identifies information on *type* and *entity*, grey on *effect*, and *blue* on *level*. Green rectangles identify the typical entity extraction performed by a NER approach.

### *Task*

The macro-task performed by our prediction model is a text-to-table task, translating a full paper abstract into a table of tuples, each one with the fields described above. Each tuple is composed itself by solving three sub-tasks:

(i) entity extraction of *mutations/variants* (from which also the *type* is inferred);
(ii) classification of *effects*;
(iii) classification of *levels*.

Tasks (ii) and (iii) are classic classification tasks, targeting a known set of values. Instead, the entity extraction task (i) is more complex than a classical Named-Entity-Recognition (NER) task: we extract mutations and variants with an associated effect and corresponding level. The complexity of this macro-task increases also because the number of tuples of the table output for each abstract is not fixed *a priori*. Instead, it depends on the number of extracted entities and on the number of effects exhibited by the entities. Text-generative models allow to fine-tune a single model that is able to perform this macro-task.

Figure 2 illustrates the working principle of our prediction task on a real abstract (Ou et al. [48]). Three different tuples are recognized in the text, all referring to the Spike V367F mutation, but predicating on different effects with higher levels. Note that the information about the protein on which the mutation occurs is positioned in a part of the text that is far apart from the signature of the mutation. In the figure, we can also appreciate the difference between the predictions obtained by our approach versus the ones that a typical NER task could obtain.

### *RegEx-based prediction filtering*

A common issue for text generative models is the instability of the generated text, i.e., these models tend to repeat words or to generate meaningless words. To mitigate this effect, we make use of a filter based on regular expressions that only allows outputs of the model corresponding to pre-defined legal values. The RegEx filter is applied after the extraction of mutations and variants to include only predictions that follow these patterns:

- Mutations: `^([A-Z0-9]+_)[A-Z]\d{1,4}[A-Z]$`
- Variants: `^([A-Z]{1,2}\.[0-9]{1,3})(\.[0-9]{1,3}){,2}$`

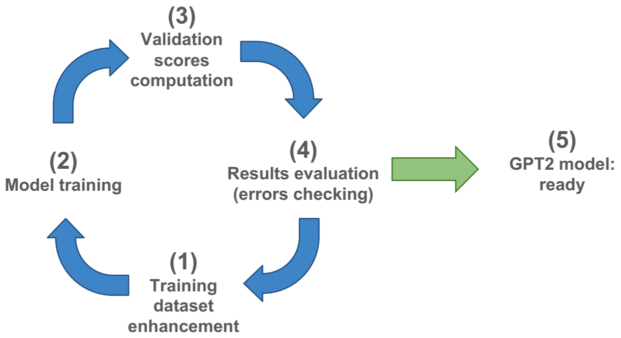


**Figure 3.** Iterative process for the prediction model setup.

## Prediction model setup

The previously described task is more complex than a classical NER task, as it requires to connect different linked information. In biomedical literature, training datasets for supervised learning are typically available for general biomedical terms [49], which are of no use for our purpose; therefore we prepared our own training dataset. This operation requires a costly manual curation, operated by highly expert users. This is an inevitable effort to handle data scarcity, analyzed in [50] in general terms, becoming even more relevant in biomedical fields [51, 52]. To minimize such effort in our case, we implemented a process that supports the bulding of small high-quality training datasets.

We started with a small number of initial abstracts (corresponding to a first set of 30 papers). Using this seed, we used an iterative process of four steps (represented in Figure 3):

(1) **Training dataset enhancement**. Except for the 1st round (30 abstracts), at each iteration we include (typically 5) new abstracts allowing stronger training on insufficiently represented cases.
(2) **Model training**. This procedure includes parameter tuning and possible changes based on previously obtained results.
(3) **Validation scores computation**. The model prediction performances are evaluated on a validation dataset of 50 papers, carefully chosen to be as representative as possible of the problem at hand. By comparing expert-provided annotations and predicted annotations on the validation dataset, we compute performance scores.
(4) **Evaluation of results and errors**. The obtained scores are considered; the iteration is repeated until satisfactory scores are obtained.

| Category | Effect | #training abs | #validation abs | #training tuples | #validation tuples |
|---|---|---|---|---|---|
| Viral kinetics and dynamics | protein_flexibility | 8 | 3 | 16 | 11 |
| | protein_stability | 29 | 3 | 47 | 9 |
| | host-virus interactions | 5 | 1 | 12 | 1 |
| | binding_to_host_receptor | 45 | 7 | 94 | 10 |
| | binding_to_antibodies | 16 | 3 | 30 | 3 |
| | viral_load | 27 | 7 | 30 | 9 |
| | viral_incubation_period | 8 | 1 | 9 | 1 |
| | viral_replication | 16 | 2 | 22 | 2 |
| | viral_fitness | 14 | 4 | 21 | 10 |
| | intermolecular_interactions | 20 | 2 | 0 | 2 |
| | protein_functioning | 19 | 2 | 32 | 5 |
| | protein_conformational_optimization | 28 | 4 | 60 | 6 |
| | entry_efficiency | 9 | 1 | 14 | 1 |
| Immunology | sensitivity_to_antibodies | 18 | 9 | 24 | 20 |
| | sensitivity_to_convalescent_sera | 20 | 6 | 32 | 10 |
| | sensitivity_to_vaccinated_sera | 20 | 6 | 35 | 11 |
| | immune_escape | 35 | 11 | 69 | 21 |
| Epidemiology | viral_transmission | 66 | 18 | 95 | 33 |
| | infectivity | 44 | 13 | 65 | 24 |
| | viral_virulence | 9 | 3 | 22 | 5 |
| | disease_severity | 32 | 8 | 62 | 15 |
| | risk_of_hospitalization | 10 | 7 | 26 | 10 |
| | risk_of_reinfection | 11 | 3 | 11 | 7 |
| | fatality_rate | 20 | 9 | 36 | 12 |
| | infection_duration | 7 | 1 | 9 | 1 |
| Diagnosis, prevention, and treatments | effectiveness_of_available_diagnostics | 13 | 1 | 23 | 1 |
| | effectiveness_of_available_vaccines | 37 | 13 | 50 | 29 |
| | effectiveness_of_available_antiviral_drugs | 23 | 6 | 50 | 10 |
| | ct_value | 12 | 2 | 14 | 2 |
| | *no relevant tuples found* | 9 | 1 | - | - |
| | **Distinct abstracts/tuples** | **221** | **50** | **1051** | **282** |

**Table 1.** Number of abstracts representing each effect in the validation and train datasets.

***Training dataset preparation***

The set of abstracts used for initial training was built by following a number of criteria:

- priority was given to published articles over preprints, excluding papers that duplicated the same research;
- priority was given to simple abstracts over abstracts with numerous and complex annotations;
- a wide selection of mutations (from different proteins) and variants (both WHO and Pangolin based names) was employed;
- abstracts involving mutations of insertion/deletion types were excluded at this stage, due to their highly heterogeneous representations;
- abstracts associating effects to groups of mutations (rather than to a single mutation or variant) were also excluded not to overly complicate the prediction task;
- no effect of our taxonomy (see AdditionalFile1-effects-taxonomy [47]) was under-represented in the dataset.

Table 1 shows comprehensive counts of abstracts containing information on each effect of our taxonomy, both for the training and the validation datasets; AdditionalFile3-training_dataset_target [47] contains the manual annotations associated with the 221 abstracts selected for training (after several iterations on the process shown in Figure 3).

***Model training***

In the iterative process the 'model training' phase is run in two different modes: 1) short-cycle training and 2) long-cycle training:

(i) *Long-cycle training* employs the whole training dataset collected thus far. It is triggered when a relevant number of annotations (60) have been collected. A manual inspection of the learning curves is conducted to performing appropriate hyperparameter tuning; the number of epochs is determined by performing an early stopping (using the validation set). When the training concludes, we generate a model freezed version (checkpoint) to be used in the following phases (validation and errors checking).

(ii) *Short-cycle training* is triggered when five new abstracts are added to the training set, aiming to update the system as soon as the new annotations are available. Here, no hyperparameters are used and the learning rate is set to half of the long-cycle training one, in order to avoid overfitting.

In both modes, the used maximum token length is 1000 and AdamW [53] is used as optimizer. The final model was trained for 12 epochs with a learning rate of $1e-5$ and a batch size of 1.

***Scores computation***

The target annotations performed by our expert researchers are available at AdditionalFile4-validation_dataset_target [47] and are supported by the text document AdditionalFile5-validation_dataset_highlighted [47] where we highlighted in yellow those information that are were used by experts to inform the annotation process and derive the target tuples.

We compare the expert annotations with the predictions of the model (see AdditionalFile6-validation_dataset_prediction [47]). Scores are computed for two different scenarios: 1) we evaluate entities, effects and levels separately (note that types are not included as they can easily be inferred from the syntax of the entity); 2) we evaluate whole tuples, including an entity with its linked effect and linked level. By comparing the target tuples – from zero to many in each abstract – with the predicted tuples, we assess the number of True Positives, False Positives, and False Negatives. Based on these observations, we compute the *accuracy*, *precision*, and *F1 score* of each abstract. Then, we obtain two aggregate scores as a simple average of the single-abstract scores (i.e., each abstract contributes equally) and a weighted average (i.e., each abstract contributes

| | | F1 score | precision | recall |
|---|---|---|---|---|
| **Entity** | average | 0,791 | 0,878 | 0,766 |
| | weighted average | 0,668 | 0,806 | 0,613 |
| **Effect** | average | 0,626 | 0,741 | 0,617 |
| | weighted average | 0,561 | 0,716 | 0,531 |
| **Level** | average | 0,762 | 0,763 | 0,761 |
| | weighted average | 0,702 | 0,705 | 0,701 |
| **Whole tuple** | average | 0,463 | 0,588 | 0,441 |
| | weighted average | 0,354 | 0,519 | 0,3 |

**Table 2.** Validation set results (run to setup the prediction model).

| | | F1 score | precision | recall |
|---|---|---|---|---|
| **Entity** | average | 0,762 | 0,802 | 0,755 |
| | weighted average | 0,688 | 0,822 | 0,822 |
| **Effect** | average | 0,792 | 0,855 | 0,781 |
| | weighted average | 0,656 | 0,656 | 0,656 |
| **Level** | average | 0,832 | 0,832 | 0,832 |
| | weighted average | 0,624 | 0,625 | 0,624 |
| **Whole tuple** | average | 0,578 | 0,631 | 0,569 |
| | weighted average | 0,324 | 0,440 | 0,288 |

**Table 3.** Test set results (run to evaluate the predictions on 100 abstracts randomly selected from the CORD-19 biology-realted cluster).

proportionally to the number of contained target tuples).

In Table 2 we show the results on the 50 papers of the validation dataset. Evaluating fields separately and using a normal average, the trained model reached 0.79 F1 score on mutation/variants, 0.63 on the effects (independently on their link to an existing entity) and 0.76 on their levels (independently on their link to an existing entity or effect). Especially for entities and effects, precision was higher than recall, indicating that the model performed well in identifying actual positives. Specifically, out of all the predicted *entities* almost 88% were actually present in the abstracts; out of all *effects*, 74% were actually present; out of all *levels* about 76% were actually present. Recall was slightly lower for entities and effects, indicating that the model missed some target information in the abstract. Specifically, recall was about 77% for *entities*, meaning that about 23% of actual entities were not recognized in abstracts; similarly, about 38% *effects* were not recognized and 24% *levels* were not recognized. Performances computed with the weighted average are generally lower, suggesting that simple abstracts (with few annotations) are the ones which contribute to improving the scores.

Finally, performances are considerably lower for the complex task of connecting the three fields in an atomic tuple (0.46 F1 score, 0.59 precision, 0.44 recall). We defend that – for such a composite task – it is more important to have higher precision (less wrong predicted annotations) at expenses of recall (missing some existing annotations). The model produces few results but in general they are of good quality. Performances can improve by augmenting the training dataset; this indeed occurs thanks to the use of the CoVEffect Web Application presented later in the manuscript.

## Results

Results include a double contribution: on the one hand we provide complete predictions on a set of more than 7 thousand abstracts from CORD-19 that are relevant to for SARS-CoV-2 variation effects; on the other hand we provide a user-friendly framework for expert users to annotate abstracts of interest and possibly contribute to additional training of the learning model.

### Annotation of the biology-related CORD-19 cluster

Abstracts informing about SARS-CoV-2 variation effects can be selected from CORD-19 via a two-steps process: 1) identification of a biology-related cluster; 2) targeted search on the cluster based on particular keywords.

*Clusters.* We built a clustering model to partition in topic-based classes the CORD-19 dataset curated by our provisioning pipeline. For this purpose, we exploited the SPECTER document-level embeddings dataset distributed as part of CORD-19 (previously described in the *similarity-based discovery*). Because of the considerable size of the dataset, we opted for a representative-based clustering model, i.e., *K-means*. SPECTER embedding vectors are known to be effective in predicting the topic class associated with a paper [36]. Differently from [36], we did not know a-priori the number of topic classes to be predicted. To choose an appropriate value for the number of clusters *k* of K-means, we plotted the silhouette score and the distortion for each candidate number of clusters, ranging from 2 to 50. The value K = 5 was chosen as it allowed to visualize a spike in the plot of the silhouette score and an elbow-like shape in the plot of the distortion. For each of the five clusters, we generated *WordCloud* plots including the most frequent words in papers' titles abstracts and titles (top words common to clusters were excluded). This allowed us to manually recognize a 100K abstracts cluster as the one mostly related to biological aspects.

*Keywords.* Out of the biology-related subset of CORD-19, we only targeted abstracts whose content relates to mutation and variants effects – the focus of CoVEffect. To this end, we described the subset of interest with a logical query expressed through the *Whoosh* search library [38] – previously mentioned for the *keyword-based search* of the data provisioning pipeline. The library already includes simple lemmatization capabilities; additionally, we loaded the OperatorsPlugin (which adds logical operators such as AND, OR, NOT), the GroupPlugin (to group search clauses using parentheses), and the SingleQuotePlugin (to specify single terms containing spaces by enclosing them in single quotes). Finally, we added a union set operation for the papers retrieved with each single query (equivalent to having all the queries in OR, but without overloading the parsing process of *Whoosh*).

As a result of this procedure – employing the keyword-based query listed in the AdditionalFile7-keywords_query_list [47], we could extract 7,230 papers from the cluster on biological aspects (see AdditionalFile8-CORD-19_batch_dataset_metadata [47]). We then ran the CoVEffect prediction on this dataset; the resulting predictions for the 7,230 abstracts are provided in AdditionalFile9-CORD-19_batch_dataset_prediction [47] as a contribution to the scientific community.

#### *Testing results*

Out of this batch, we tested the prediction performances on 100 randomly selected papers, ensuring that they did not overlap with the previously used training and validation sets. For these, we manually prepared target annotations (see AdditionalFile10-test_dataset_target [47]). Then, we predicted the annotations of their abstracts using our model (see AdditionalFile11-test_dataset_prediction [47]). In Table 3, we show the results on the 100 papers of the test dataset, based on the comparison between target and predicted annotations. Reassuringly, performances were comparable to the ones obtained on the validation set. Indeed, they were only worse in the case of *entities*, whereas *effects*, *levels*, and also whole tuples, improved their scores.

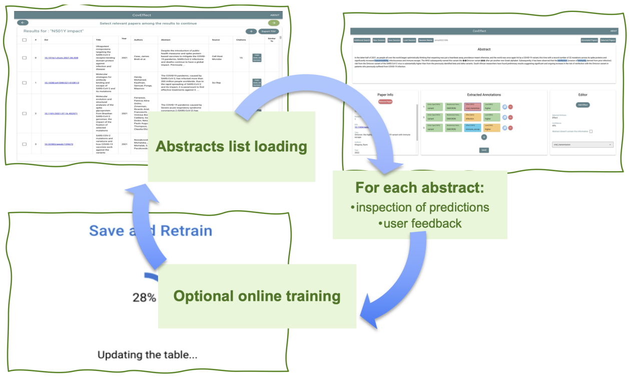


**Figure 4.** The three iterative phases of the online semi-automated data labeling framework.

## The CoVEffect Web application

As a second output, we implemented the CoVEffect Web application; its front-end provides two main functionalities: 1) a search interface for finding papers of interest; and 2) an interactive interface to label abstracts with a semi-automated framework. The first functionality is based on a back-end retrieval module, which uses the methods described in the 'Data provisioning' section (i.e., keyword based search and similarity-based search of papers). The second functionality is fuelled by a back-end extraction module, which uses the prediction model described in the 'Language model and task design' section and implements a framework for semi-automated data labeling by users, as detailed in the following.

### *Semi-automated data labeling framework*

This framework aims to facilitate and accelerate the abstract annotation process operated by an expert researcher. We can describe a typical annotation session with iterative phases, as shown in Figure 4:

- The user provides a list of abstracts.
- For each selected abstract: 1) the model generates a proposed labeling in the form of predicted tuples; 2) the user may edit each single prediction (i.e., one tuple field at a time).
- Once the editing session is over, the user is provided the choice of accepting the annotations and of re-training the model with the new provided annotations.

The user may modify or add abstracts to the list at any point in time. For each prediction (type, entity, effect, or level), the framework provides two types of visual feedback. First, it shows the prediction confidence value with a color code: *green* for high confidence predictions > 0.8, *yellow* for medium-confidence predictions between 0.6 and 0.8, and *red* for low-confidence predictions < 0.6. Second, it shows a saliency map built on the input abstract. Saliency maps are a machine learning interpretation mechanism born in the field of EXplainable AI; they are maps over the input that highlight the portions of the text that contributed the most to the extraction of given attributes. Here, we exploited the generation of saliency maps that employ the Gradient technique (Atanasova et al. [54]). Such idea was already proposed successfully in our previous work [29] where such mechanism was well-evaluated by the users of the system, as it allow users to understand whether a given result is not only predicted correctly, but also predicted by exploiting a correct information. As an example, in Figure 5 we show the saliency map obtained for the prediction of the 'infectivity' effect on the abstract of Ou et al. [48] previously introduced in Figure 2.

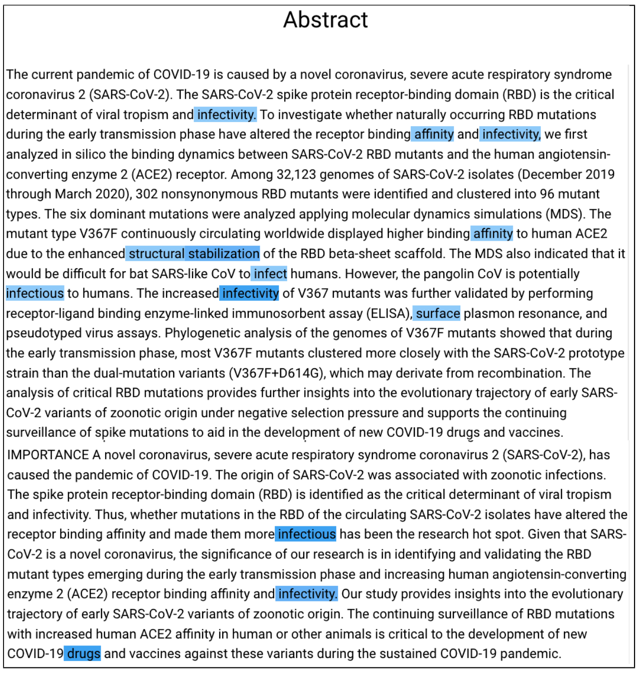
Abstract

The current pandemic of COVID-19 is caused by a novel coronavirus, severe acute respiratory syndrome coronavirus 2 (SARS-CoV-2). The SARS-CoV-2 spike protein receptor-binding domain (RBD) is the critical determinant of viral tropism and infectivity. To investigate whether naturally occurring RBD mutations during the early transmission phase have altered the receptor binding affinity and infectivity, we first analyzed in silico the binding dynamics between SARS-CoV-2 RBD mutants and the human angiotensin-converting enzyme 2 (ACE2) receptor. Among 32,123 genomes of SARS-CoV-2 isolates (December 2019 through March 2020), 302 nonsynonymous RBD mutants were identified and clustered into 96 mutant types. The six dominant mutations were analyzed applying molecular dynamics simulations (MDS). The mutant type V367F continuously circulating worldwide displayed higher binding affinity to human ACE2 due to the enhanced structural stabilization of the RBD beta-sheet scaffold. The MDS also indicated that it would be difficult for bat SARS-like CoV to infect humans. However, the pangolin CoV is potentially infectious to humans. The increased infectivity of V367 mutants was further validated by performing receptor-ligand binding enzyme-linked immunosorbent assay (ELISA), surface plasmon resonance, and pseudotyped virus assays. Phylogenetic analysis of the genomes of V367F mutants showed that during the early transmission phase, most V367F mutants clustered more closely with the SARS-CoV-2 prototype strain than the dual-mutation variants (V367F+D614G), which may derivate from recombination. The analysis of critical RBD mutations provides further insights into the evolutionary trajectory of early SARS-CoV-2 variants of zoonotic origin under negative selection pressure and supports the continuing surveillance of spike mutations to aid in the development of new COVID-19 drugs and vaccines.
IMPORTANCE A novel coronavirus, severe acute respiratory syndrome coronavirus 2 (SARS-CoV-2), has caused the pandemic of COVID-19. The origin of SARS-CoV-2 was associated with zoonotic infections. The spike protein receptor-binding domain (RBD) is identified as the critical determinant of viral tropism and infectivity. Thus, whether mutations in the RBD of the circulating SARS-CoV-2 isolates have altered the receptor binding affinity and made them more infectious has been the research hot spot. Given that SARS-CoV-2 is a novel coronavirus, the significance of our research is in identifying and validating the RBD mutant types emerging during the early transmission phase and increasing human angiotensin-converting enzyme 2 (ACE2) receptor binding affinity and infectivity. Our study provides insights into the evolutionary trajectory of early SARS-CoV-2 variants of zoonotic origin. The continuing surveillance of RBD mutations with increased human ACE2 affinity in human or other animals is critical to the development of new COVID-19 drugs and vaccines against these variants during the sustained COVID-19 pandemic.

**Figure 5.** The gradient based saliency map implemented in the CoVEffect tool. The example shows the abstract of the paper by Ou et al. [48] also used in Figure 2 to motivate our task. The text fragments highlighted with different shades of blue are used by the model to predict the effect of the SPIKE_V367F mutation, here corresponding to the value 'infectivity'.

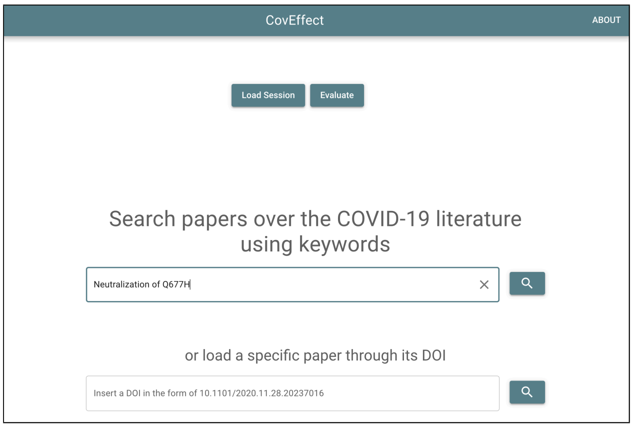


**Figure 6.** Home page, with a section for keyword search and a section for DOI search.

### *Application workflow and example*

The 'Home page' of CoVEffect accepts two kinds of input, i.e., a list of keywords or a single DOI. Suppose that we search for the keywords 'Neutralization of Q677H' (as shown in Figure 6). The following workflow is explained by the activity diagram in Figure 7.

Once the search is performed, we reach the 'Search result page', whose results can be examined (based on their metadata and abstract) and exported as a tab-separated file. Extracted papers may be of interest for the user (especially when they are focused on mutations or variants effects), in which case they can be included in the prediction stack. For each paper, users may also explore similar papers, by opening the 'Similar papers tab'; as before, papers of interest, can be selected. When the user closes the tab, she will have a complete list of the searched papers, where papers selected are marked in grey and papers added for the similar ones are marked in green. Figure 8 shows an example where, from the papers obtained in the previous search, we selected the paper with DOI '10.1128/mbio.02510-21' [55] and its similar paper with DOI '10.1186/s12985-021-01554-8' [56].

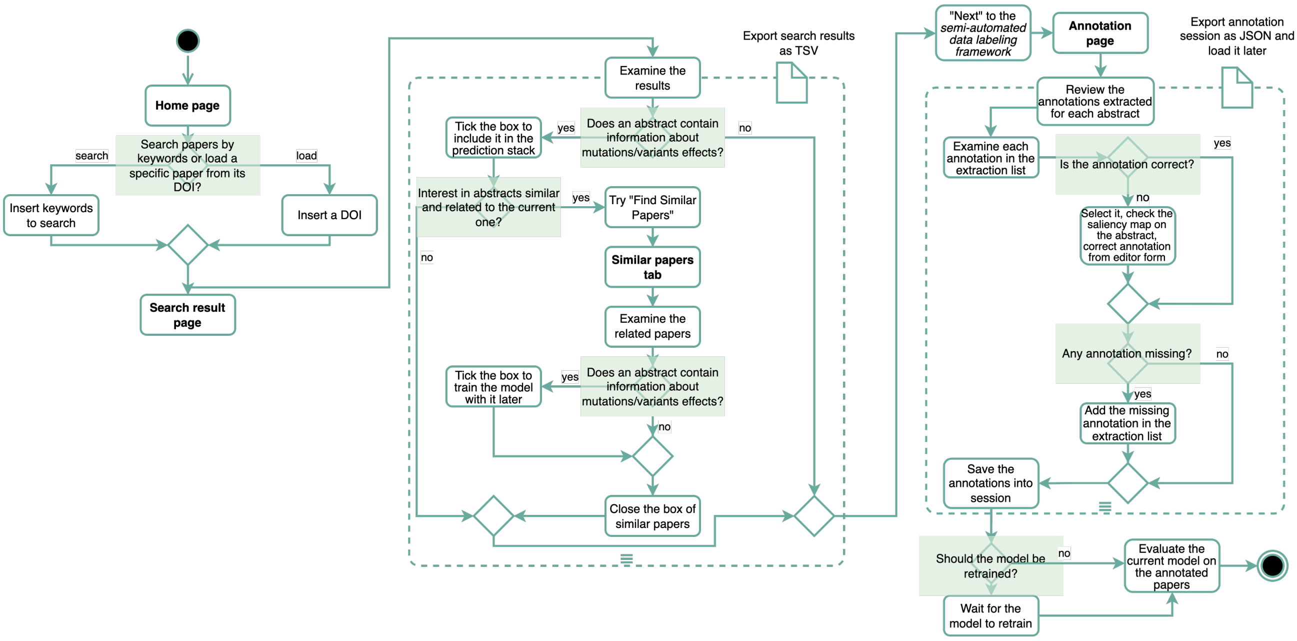


**Figure 7.** Activity diagram of the user's interactions with CoVEffect Web application.

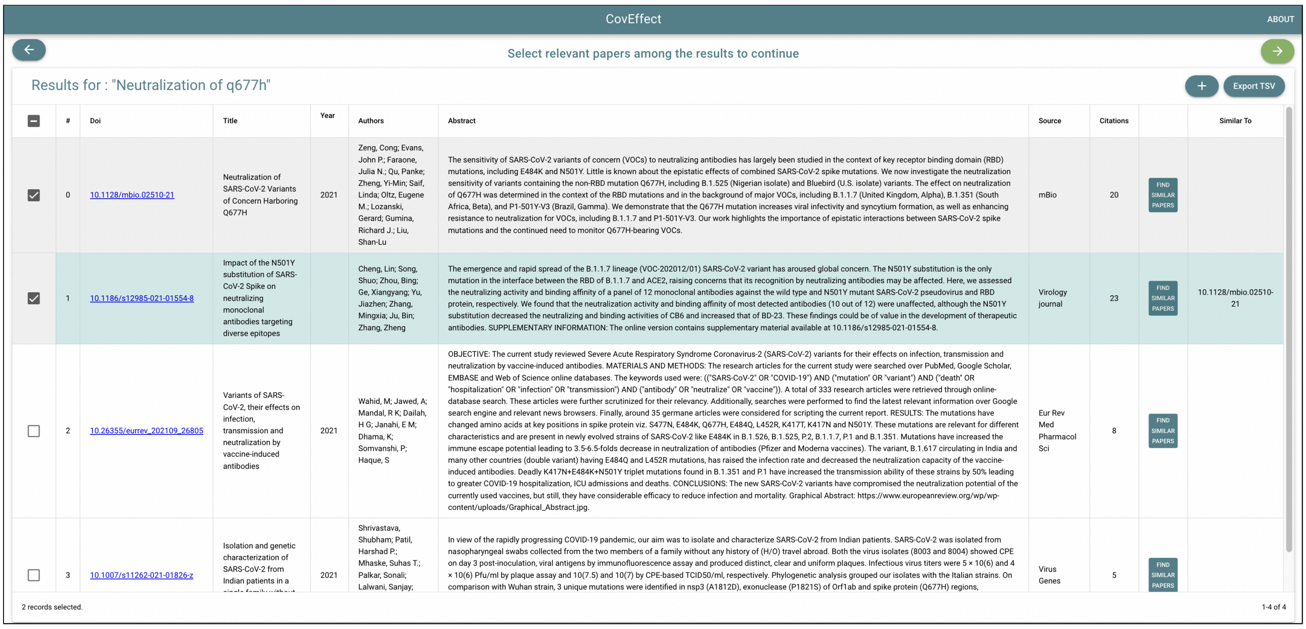


**Figure 8.** Paper List screen, obtained after searching for 'Neutralization of Q677H' and inspecting papers similar to the first one (DOI '10.1128/mbio.02510-21' [55]). Papers that are selected by the user are highlighted in color: grey for the ones corresponding to the initial search, green for the ones corresponding to the similarity-based search.

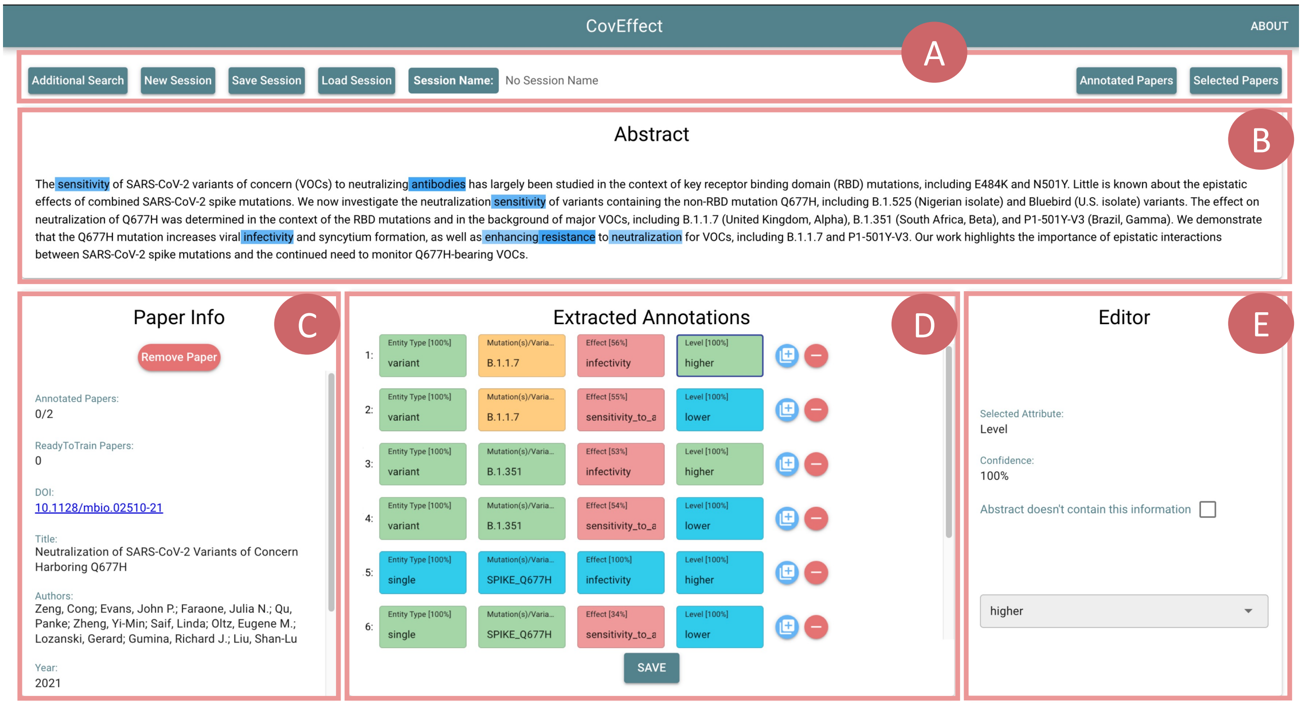


**Figure 9.** Overview of the CoVEffect interface, with a top bar and four panels, captured during the annotation of a paper by Zeng et al. [55], `https://doi.org/10.1128/mbio.02510-21`. Panel A includes the top bar; the commands on the left allow to return to the key-word search screen, open a new user session, save the current one or load a previously closed one. The commands on the right allow to inspect the list of already processed papers or the list of papers selected through the keyword search). Panel B shows the abstract of the selected paper to be annotated, interactively highlighted using the gradient-based saliency map related to the tuple fragment selected in Panel D. Panel C shows the metadata of the selected paper and the size of the stack of papers chosen by the user. Panel D shows the predicted tuples for the selected abstract, using the color-code for informing on the accuracy of the prediction. Panel E allows users to actively modify the prediction of the model and save the suggestions.

By pressing the green arrow on the top right corner of the screen, we reach the 'Annotation page'. From this page, users are allowed to inspect results and suggest changes for one abstract at a time. For each abstract, the framework extracts a list of predicted tuples, each composed of four fields (type, entity, effect, and level). For each of such annotations, the user can inspect the saliency map, decide if the annotation is correct (thus should be approved) or needs correction. Missing annotations can also be added manually.

Figure 9 represents the status of the 'Annotation page' for paper [55]. Panel A provides user utilities. Panel B shows the saliency map referring to the prediction of the value 'higher' for the level of the first predicted tuple (selected in Panel D). Panel C shows the metadata of the currently inspected paper and informs that the prediction stack contains 2 papers (of which none has yet been annotated, as we have not clicked on 'SAVE'). Panel D shows predictions 1, 2, 3, 4, and 6 as produced by the prediction framework, with the exception of the level values of 2, 4, and 6 that have been manually corrected into the 'lower' value (which had been wrongly predicted), by employing the drop-down menu in Panel E.

In addition, a full tuple annotation has been added (number 5) regarding the single mutation Spike Q677H, which leads to an increase in infectivity of the SARS-CoV-2 virus.

When the user is satisfied with all the annotations associated to an abstract, these can be saved and are accordingly stored in the 'Annotated Papers' list (Panel A, top right corner), where they can also be downloaded for further processing. Note that annotated abstracts that can be saved are the result of either a model prediction or of a user manual correction/addition.

When saving annotations for the first time, the user is prompted to name the current session. Sessions can be downloaded as JSON files and reloaded at a later time. Then, the user is asked if she wishes to retrain the model immediately. This process is computationally intensive and may require several minutes based on the occupation of the servers. Users may also wait to annotate additional papers and retrain the model only at a later stage. The application can be installed on other machines using the Docker distribution available on our GitHub repository.

## Discussion

In this paper we described a duple contribution. On one hand we provide the identification of SARS-CoV-2 variants and mutations' effects over a relevant set of CORD-19 abstracts. On the other hand, we make this annotation extendable, as training data can be augmented by using the CoVEffect interface. The project stems from the need of providing a complete framework that supports semi-automatic extraction of structured information on SARS-CoV-2 variation effects. We had previously employed transformer-based text extraction for capturing key-value pairs from genomic experiments (from Gene Expression Omnibus). The task performed in this case is more complex, as it aims to identify attributes that are inter-dependent: mutation or variants with their effect and level.

A considerable improvement of the initial GPT2 model was necessary to address this new challenge. In addition, no pre-existing training dataset was available; we thus designed a methodology to build a small manually crafted dataset of good quality. The trajectory to evaluate the performances of our method is as follows: we chose an initial dataset with minimal size, at each small delta increase we evaluate the changes in performances on a test dataset until a satisfactory result is reached. This process was necessary to find a trade-off between two needs: the minimization of the effort of expert manual annotation and maximization of prediction performances. This effort has payed off in terms of recognizing single concepts; however the linked tuple prediction still has much space of improvement.

To inspect the most challenging aspects of the prediction task, we performed an error analysis divided into three categories: 1) entity name prediction (non-constrained to any value, filtered with a RegEx filter); 2) effect/level prediction (restricted to our taxonomy

| | Issue | DOI | Text excerpt from abstract | Target | Prediction |
|---|---|---|---|---|---|
| Entity name prediction | Uncommon naming (mutations/variants) | [57] | The S:655Y substitution was transmitted more efficiently than [...] | SPIKE_H655Y | - |
| | Mutations/variants referred to as a group | [55] | [...] major VOCs, including Alpha, Beta, and Gamma. We demonstrate that the Q677H mutation increases viral infectivity and syncytium formation, as well as enhancing resistance to neutralization for VOCs. | Alpha | Alpha |
| | | | | Beta | Beta |
| | | | | Gamma | - |
| | Mutations/variants reported as long lists | [58] | To understand the impact of spike protein mutations on the binding interactions required for virus infection and the effectiveness of neutralizing monoclonal antibody (mAb) therapies, mutants D614G, N501Y, N439K, Y453F, and E484K were assessed. | SPIKE_ D614G | - |
| | | | | SPIKE_N501Y | SPIKE_N501Y |
| | | | | SPIKE_N439K | - |
| | | | | SPIKE_Y453F | SPIKE_Y453F |
| | | | | SPIKE_E484K | SPIKE_E484K |
| Effect and/or level prediction | Effect terminology | [59] | The increased ACE2-binding affinity of variants containing the N501Y or E484K mutations can be traced to the time-dependent disruption and/or formation of interfacial salt bridges, not necessarily apparent from structural models but detected by extensive molecular dynamics simulations. | SPIKE_N501Y binding to host receptor | SPIKE_N501Y binding to host receptor |
| | | | | SPIKE_N501Y intermolecular interactions | SPIKE_N501 protein conformational opt. |
| | | | | SPIKE_E484K binding to host receptor | SPIKE_E484K binding to host receptor |
| | | | | SPIKE_E484K intermolecular interactions | SPIKE_E484 protein conformational opt. |
| | Vague results presentation | [57] | We demonstrate that the substitution S:655Y, represented in the Gamma and Omicron VOCs, enhances viral replication and spike protein cleavage. All VOCs tested exhibited increased spike cleavage and fusogenic capacity. | GAMMA viral replication | GAMMA viral replication |
| | | | | GAMMA protein functioning | - |
| Association | Multiple effects connected to same entity | [60] | Infections caused by the delta variant increases the risk of hospitalization within 14 days after symptom onset, and the high viral load correlates with COVID-19 associated morbidity and mortality. | DELTA, risk of hospitaliz. | DELTA, risk of hospitaliz. |
| | | | | DELTA, viral load | DELTA, viral load |
| | | | | DELTA, fatality rate | - |
| | Different levels for same entity-effect (not supported) | [61] | Naturally occurring variants in Orf3a (Q57H) and nsp2 (T85I) were associated with poor replication in Vero-CCL81 cells but not in BEpCs. | ORF3a_Q57H viral replication, lower | - |
| | | | | ORF3a_Q57H viral replication, unaffected | ORF3a_Q57H viral replication, unaffected |

**Table 4.** Typical issues detected in the prediction task. The first column groups issues by macro-category, the second describes the scenario that leads to an *Issue*, the third and forth provide the reference **DOI** to an abstract and a short **Text excerpt from the abstract**. Yellow text highlighting is used to show the relevant information for the expected values (**Target**) as opposed to the obtained **Prediction**.

values); and 3) association between entity, effect, and level. Table 4 presents an overview of the most representative errors each with an associated example.

Types of errors captured in the *entity name prediction* mainly occurred when the abstract includes:

- *Mutation/variant named with uncommon terminology.* The typical way to name a mutation is to declare the protein where the mutation occurred followed by a mutation signature (⟨reference amino acid, coordinate in protein, alternative amino acid⟩, e.g., Spike D614G). The most adopted terminologies to name a SARS-CoV-2 variant are Pango lineages [45] or WHO Greek-letters [20]; however, there are other ways to referring to variants (e.g., GISAID or Nextstrain clades), which are currently not supported in CoVEffect. Table 4 shows an example from [57] where a different naming scheme is used for a mutation of interest, which makes the model's mission harder.
- *Effect/level associated with a named group of variants.* The WHO has classified variants into Variants of Concern (VoCs) and other classes according to their impacts [20]. In publications we often find reference to effects studied on a group of variants, referred to with such terms. Table 4 shows one such case [55], where CoVEffect can miss one or more entities in the list.
- *Mutations/variants written as long lists.* Some publications – noticeably the ones using computational methods to analyze their variants of interest – tend to deal with long lists of mutations. CoVEffect model may miss some entities in such scenarios (as it happened in [58]).

Moreover, issues occurring in the *entity/level prediction* mainly occurred when the abstract includes:

- *Effects mis-classification.* The model does not always recognize effects as they are expressed in our taxonomy, especially when there exist connections between different effects. This case may happen when an effect is a special case of another effect, e.g., binding to host receptor is a special case of a host-virus interaction; in this case, only using a broad context and expert

user knowledge it becomes possible to understand the correct target effect. Table 4 shows one such example from [59].

- *Levels mis-classification.* The changes of some effects are more easily expressible through the higher/lower comparators (i.e., higher transmissibility, lower severity). Unfortunately for other effects (e.g., protein conformational optimization) comparators are less used in text.
- *Unclear results presentation.s* Effects reported in abstracts with a vague presentation of the results can be missed. For example, some publications that report on the effectiveness of a specific therapeutic measure, could exempt from declaring that the measure is indeed a drug. Other publications (see [57] for an example) study the effect of a mutation on the functions of viral proteins without making explicit that topic discussed is protein function – making it hard for the model to predict the effect.

Finally, problems occurring in predicting the *association between an entity and its effect-level* mainly occurred when the abstract includes:

- *Multiple effects for one entity.* The model can miss the association of one (or more) effects that are part of a list (as it happens for [60]).
- *Multiple levels for one entity-effect.* Given abstracts may include the specification of an entity and associated effect with multiple levels (e.g., in [61]). This scenario is likely to be found when the specific effect has been studied under multiple conditions, e.g., measuring the viral loads of a variant in different tissues or studying the binding of a specific variant with a wide range of antibodies. CoVEffect current data model does not support multiple disagreeing levels for an entity-effect pair. This impacts on the recall (w.r.t. precision) of our results.

Notably, the prediction model reached quite good performances as shown in Tables 2-3 and still has much space for improvement thanks to the expected enhancements on the training dataset. An interesting result is that mutation entities were very well predicted even when the protein information was far apart in the text from the mutation signature (see our motivating example in Figure 2 where Spike is far from V367F but they are correctly associated); the interpretability mechanism of saliency maps is of great support to highlight these cases. Moreover, the model worked well in detecting our targets: protein amino acid-based mutations rather than genomic nucleotide-based mutations and lineages rather than clades.

CoVEffect brings a number of tangible results to the scientific community, that we here describe. Immediate integrated use of our resulting annotated database was made within our CoV2K [22] system, by updating the AA_changes, Variant, and Effect entities.

Others data-driven analysis resources developed by our group (such as VirusViz [62] and ViruClust [63]) could immediately benefit from the addition of structured tuples connecting mutations and effects At the same time, any other resource employed in the current practice of virologists and phylogenetists (such as CoV-Spectrum [64] and Outbreak.info [65]), studying the trend of specific mutations and variants, can benefit from the provisioning of a dataset with this structured information. Our output can be appreciated in the AdditionalFile9 [47], containing the predicted annotations for the whole biology-related CORD-19 cluster. External users may also annotate other abstracts by installing CoVEffect through our Docker distribution and running the batch annotator (available as a Python notebook on our GitHub repository).

As future directions, we aim to extend the scope of CoVEffect by including the possibility of recognizing also: alternative formulations of mutation and variant names; tuples reporting on different levels for the same entity and effect; groups of mutations leading collaboratively to the same effect; insertions and deletions; the method used to establish the effect (epidemiological, experimental, computational or inferred); effects reported with complex – possibly quantitative – formulations.

In the future, we aim to apply CoVEffect to other subparts of the CORD-19 dataset as well as to expand to other literature corpuses, focusing on different, well-defined and delimited domains. More in general, our framework is suitable to resolve similar problems where the prediction task attempts to recognize in text the associations between given entities and related values (within existing taxonomies).

## Availability of Source Code and Requirements

The source code corresponding to the data provisioning module, the deep learning-based prediction framework, and the CoVEffect web interface to annotate abstracts are available in the GitHub repository https://github.com/armando2603/coveffect.

## Data Availability

The datasets and all 'Additional Files' supporting the results of this article are available in the repository [47].

## Additional Files

**AdditionalFile1-effects-taxonomy:** Descriptions of legal values for the 'Effect' field, based on a categorized taxonomy.
**AdditionalFile2-levels-taxonomy:** Descriptions of legal values for the 'Level' field.
**AdditionalFile3-training_dataset_target:** List of target tuples (manually annotated) of 221 abstracts considered for training the model. For each abstract, target tuples follow the schema ID, DOI, title, entity, effect, level, type (mutation or variant), tuples_count (>1 when an effect/level is shared by multiple entities, #abstracts containing the same effect described in the tuple).
**AdditionalFile4-validation_dataset_target:** List of target tuples (manually annotated) of 50 abstracts considered for validating the prepared prediction model. For each abstract, target tuples follow the schema defined for AdditionalFile3.
**AdditionalFile5-validation_dataset_highlighted:** Textual abstracts of the 50 manuscripts considered for validation; the text used to support the manual target annotations has been highlighted in yellow.
**AdditionalFile6-validation_dataset_prediction:** List of predicted annotations of 50 abstracts considered for validating the prepared prediction model; it contains 4 sheets, respectively for entity, effect, level, and whole tuple predictions.
**AdditionalFile7-keywords_query_list:** Keyword-based search run on the CORD-19 dataset to extract a relevant subset of abstracts regarding the scope of interest of CoVEffect. The Boolean logic used to combine keywords is explained in the section 'Annotations of the biology-related CORD-19 cluster'.
**AdditionalFile8-CORD-19_batch_dataset_metadata:** Metadata of the 7,230 papers extracted by the keyword-based query in AdditionalFile7. These abstracts have been annotated by the prediction framework.
**AdditionalFile9-CORD-19_batch_dataset_prediction:** List of predicted annotations of 7,230 abstracts extracted from the biology-related cluster of CORD-19.
**AdditionalFile10-test_dataset_target:** List of target tuples (manually annotated) of 100 abstracts randomly selected from the 7,230 extracted as in AdditionalFile8. For each abstract, target tuples follow the schema defined for AdditionalFile3.
**AdditionalFile11-test_dataset_prediction:** List of predicted annotations of 100 abstracts considered for testing the prediction model on a subset of the CORD-19 biology-related cluster. As AdditionalFile6,

it contains 4 sheets, respectively for entity, effect, level, and whole tuple predictions.

# Declarations

## List of abbreviations

CDC: Centers for Disease Control and Prevention;
CORD-19: COVID-19 Open Research Dataset;
ECDC: European Centre for Disease Prevention and Control;
GPT2: Generative Pre-trained Transformer 2;
NER: Named Entity Recognition;
NLP: Natural Language Processing;
SARS-CoV-2: Severe Acute Respiratory Syndrome CoronaVirus 2;
WHO: World Health Organization.

## Ethical approval

Not applicable.

## Consent for publication

Not applicable.

## Competing interests

The authors declare that they have no competing interests.

## Funding

CoVEffect is supported by the ERC Advanced Grant 693174 "Data-Driven Genomic Computing (GeCo)" and by the grant "Amazon Machine Learning Research Awards". The funders had no role in study design, data collection and analysis, decision to publish, or preparation of the manuscript.

## Author's contributions

G.G.S.: Formal Analysis, Investigation, Methodology, Software (back-end prediction model, front-end, evaluation).
R.A.K.: Data curation, Investigation, Validation.
F.I.: Methodology, Software (data provisioning, keyword search), Validation.
S.C.: Funding acquisition, Writing – review & editing.
A.B.: Conceptualization, Project administration, Supervision, Visualization, Writing – original draft.

# Acknowledgements

The authors would like to thank Mark J. Carman for inspiring the first prototype of the semi-automated data labeling framework and Giuseppe Cannizzaro for building the first prototype of the transformer-based prediction model.

# References

1. Else H. How a torrent of COVID science changed research publishing–in seven charts. Nature 2020;588(7839):553–554.
2. Wang LL, Lo K, Chandrasekhar Y, Reas R, Yang J, Burdick D, et al. CORD-19: The COVID-19 Open Research Dataset. In: Proceedings of the 1st Workshop on NLP for COVID-19 at ACL 2020 Online: Association for Computational Linguistics; 2020. https://www.aclweb.org/anthology/2020.nlpcovid19-acl.1.
3. Shu Y, McCauley J. GISAID: Global initiative on sharing all influenza data–from vision to reality. Eurosurveillance 2017;22(13).
4. Sayers EW, Cavanaugh M, Clark K, Pruitt KD, Sherry ST, Yankie L, et al. GenBank 2023 update. Nucleic Acids Research 2022;Gkac1012.
5. The COVID-19 Genomics UK (COG-UK) consortium. An integrated national scale SARS-CoV-2 genomic surveillance network. The Lancet Microbe 2020;1(3):e99.
6. Bernasconi A, Mari L, Casagrandi R, Ceri S. Data-driven analysis of amino acid change dynamics timely reveals SARS-CoV-2 variant emergence. Scientific Reports 2021;11(1):1–10.
7. Chiara M, Horner DS, Gissi C, Pesole G. Comparative genomics reveals early emergence and biased spatiotemporal distribution of SARS-CoV-2. Molecular biology and evolution 2021;38(6):2547–2565.
8. Huang Q, Zhang Q, Bible PW, Liang Q, Zheng F, Wang Y, et al. A new way to trace SARS-CoV-2 variants through weighted network analysis of frequency trajectories of mutations. Frontiers in Microbiology 2022;13.
9. Korber B, Fischer WM, Gnanakaran S, Yoon H, Theiler J, Abfalterer W, et al. Tracking changes in SARS-CoV-2 spike: evidence that D614G increases infectivity of the COVID-19 virus. Cell 2020;182(4):812–827.
10. Hodcroft EB, Zuber M, Nadeau S, Vaughan TG, Crawford KH, Althaus CL, et al. Spread of a SARS-CoV-2 variant through Europe in the summer of 2020. Nature 2021;595(7869):707–712.
11. Li Q, Wu J, Nie J, Zhang L, Hao H, Liu S, et al. The impact of mutations in SARS-CoV-2 spike on viral infectivity and antigenicity. Cell 2020;182(5):1284–1294.
12. Radford A, Wu J, Child R, Luan D, Amodei D, Sutskever I, et al. Language models are unsupervised multitask learners. OpenAI blog 2019;1(8):9.
13. Rophina M, Pandhare K, Mangla M, Shamnath A, Jolly B, Sethi M, et al. FaviCoV-a comprehensive manually curated resource for functional genetic variants in SARS-CoV-2. OSF Preprints 2020;.
14. Rophina M, Pandhare K, Shamnath A, Imran M, Jolly B, Scaria V. ESC: a comprehensive resource for SARS-CoV-2 immune escape variants. Nucleic acids research 2022;50(D1):D771–D776.
15. Wright DW, Harvey WT, Hughes J, Cox M, Peacock TP, Colquhoun R, et al. Tracking SARS-CoV-2 mutations and variants through the COG-UK-Mutation Explorer. Virus Evolution 2022;8(1):veac023.
16. Subissi L, von Gottberg A, Thukral L, Worp N, Oude Munnink BB, Rathore S, et al. An early warning system for emerging SARS-CoV-2 variants. Nature medicine 2022;p. 1–6.
17. Torrens-Fontanals M, Peralta-García A, Talarico C, Guixà-González R, Giorgino T, Selent J. SCoV2-MD: a database for the dynamics of the SARS-CoV-2 proteome and variant impact predictions. Nucleic acids research 2022;50(D1):D858–D866.
18. Hodcroft EB, CoVariants: SARS-CoV-2 Mutations and Variants of Interest;. (2021). Last accessed: December 5th, 2022. https://covariants.org/.
19. European Centre for Disease Prevention and Control, SARS-CoV-2 variants of concern; 2021. Last accessed: December 5th, 2022. https://www.ecdc.europa.eu/en/covid-19/variants-concern.
20. World Health Organization, Tracking SARS-CoV-2 variants;. (2021). Last accessed: December 5th, 2022. https://www.who.int/en/activities/tracking-SARS-CoV-2-variants/.
21. Centers for Disease Control and Prevention, SARS-CoV-2 Variant Classifications and Definitions; 2022. Last accessed: December 5th, 2022. https://www.cdc.gov/coronavirus/2019-ncov/variants/variant-info.html.
22. Alfonsi T, Al Khalaf R, Ceri S, Bernasconi A. CoV2K model, a comprehensive representation of SARS-CoV-2 knowledge and

data interplay. Scientific Data 2022;9:260.
23. Lan K, Wang Dt, Fong S, Liu Ls, Wong KK, Dey N. A survey of data mining and deep learning in bioinformatics. Journal of medical systems 2018;42:139.
24. Clauwaert J, Menschaert G, Waegeman W. Explainability in transformer models for functional genomics. Briefings in Bioinformatics 2021 04;22(5). Bbab060.
25. Kalyan KS, Rajasekharan A, Sangeetha S. AMMU: A Survey of Transformer-based Biomedical Pretrained Language Models. Journal of Biomedical Informatics 2021;p. 103982.
26. Warikoo N, Chang YC, Hsu WL. LBERT: Lexically aware Transformer-based Bidirectional Encoder Representation model for learning universal bio-entity relations. Bioinformatics;37(3):404–412.
27. Lai PT, Lu Z. BERT-GT: cross-sentence n-ary relation extraction with BERT and Graph Transformer. Bioinformatics;36(24):5678–5685.
28. Cannizzaro G, Leone M, Bernasconi A, Canakoglu A, Carman MJ. Automated integration of genomic metadata with sequence-to-sequence models. In: Joint European Conference on Machine Learning and Knowledge Discovery in Databases Springer; 2020. p. 187–203.
29. Serna Garcia G, Leone M, Bernasconi A, Carman MJ. GeMI: interactive interface for transformer-based Genomic Metadata Integration. Database 2022;2022.
30. Barrett T, Wilhite SE, Ledoux P, Evangelista C, Kim IF, Tomashevsky M, et al. NCBI GEO: archive for functional genomics data sets–update. Nucleic acids research 2012;41(D1):D991–D995.
31. He H, Fu S, Wang L, Liu S, Wen A, Liu H. MedTator: a serverless annotation tool for corpus development. Bioinformatics 2022;38(6):1776–1778.
32. Feng Y, Qi L, Tian W. PhenoBERT: a combined deep learning method for automated recognition of human phenotype ontology. IEEE/ACM Transactions on Computational Biology and Bioinformatics 2022;.
33. Balabin H, Hoyt CT, Birkenbihl C, Gyori BM, Bachman J, Kodamullil AT, et al. STonKGs: a sophisticated transformer trained on biomedical text and knowledge graphs. Bioinformatics 2022;38(6):1648–1656.
34. Balabin H, Hoyt CT, Gyori BM, Bachman JA, Kodamullil AT, Hofmann-Apitius M, et al. ProtSTonKGs: A Sophisticated Transformer Trained on Protein Sequences, Text, and Knowledge Graphs. In: SWAT4HCLS; 2022. p. 103–107.
35. Mahajan D, Liang JJ, Tsou CH. Toward Understanding Clinical Context of Medication Change Events in Clinical Narratives. In: AMIA Annual Symposium Proceedings, vol. 2021 American Medical Informatics Association; 2021. p. 833.
36. Cohan A, Feldman S, Beltagy I, Downey D, Weld DS. SPECTER: Document-level Representation Learning using Citation-informed Transformers. arXiv 2020;https://arxiv.org/abs/2004.07180.
37. Danilák M, Port of Nakatani Shuyo's language-detection library to Python; 2022. Last accessed: December 5th, 2022. https://github.com/Mimino666/langdetect.
38. Chaput M, Whoosh search engine library; 2022. Last accessed: December 5th, 2022. https://github.com/mchaput/whoosh.
39. Spotify, Annoy (Approximate Nearest Neighbors Oh Yeah); 2022. Last accessed: December 5th, 2022. https://github.com/spotify/annoy.
40. Devlin J, Chang M, Lee K, Toutanova K. BERT: Pre-training of Deep Bidirectional Transformers for Language Understanding. arXiv 2018;http://arxiv.org/abs/1810.04805.
41. Zhu Y, Kiros R, Zemel RS, Salakhutdinov R, Urtasun R, Torralba A, et al. Aligning Books and Movies: Towards Story-like Visual Explanations by Watching Movies and Reading Books. arXiv 2015;http://arxiv.org/abs/1506.06724.
42. Romero M, GPT-2-finetuned-CORD19;. Last accessed: December 5th, 2022. https://huggingface.co/mrm8488/GPT-2-finetuned-CORD19.
43. Wu F, Zhao S, Yu B, Chen YM, Wang W, Song ZG, et al. A new coronavirus associated with human respiratory disease in China. Nature 2020;579(7798):265–269.
44. Lauring AS, Hodcroft EB. Genetic variants of SARS-CoV-2—what do they mean? Jama 2021;325(6):529–531.
45. Rambaut A, Holmes EC, O'Toole Á, Hill V, McCrone JT, Ruis C, et al. A dynamic nomenclature proposal for SARS-CoV-2 lineages to assist genomic epidemiology. Nature Microbiology 2020;5(11):1403–1407.
46. Al Khalaf R, Alfonsi T, Ceri S, Bernasconi A. CoV2K: A Knowledge Base of SARS-CoV-2 Variant Impacts. In: Cherfi S, Perini A, Nurcan S, editors. Research Challenges in Information Science Cham: Springer International Publishing; 2021. p. 274–282.
47. Serna Garcia G, Al Khalaf R, Invernici F, Ceri S, Bernasconi A, Supporting data for "CoVEffect: Interactive System for Mining the Effects of SARS-CoV-2 Mutations and Variants Based on Deep Learning"; 2022. [*temporarily hosted on* https://tinyurl.com/additionalfilesCoVEffect, *to be changed on acceptance of manuscript*].
48. Ou J, Zhou Z, Dai R, Zhang J, Zhao S, Wu X, et al. V367F mutation in SARS-CoV-2 spike RBD emerging during the early transmission phase enhances viral infectivity through increased human ACE2 receptor binding affinity. Journal of virology 2021;95(16):e00617–21.
49. Wang B, Xie Q, Pei J, Tiwari P, Li Z, Fu J. Pre-trained Language Models in Biomedical Domain: A Systematic Survey. arXiv 2021;https://arxiv.org/abs/2110.05006.
50. Bansal MA, Sharma DR, Kathuria DM. A systematic review on data scarcity problem in deep learning: solution and applications. ACM Computing Surveys (CSUR) 2022;54(10s):1–29.
51. Tinn R, Cheng H, Gu Y, Usuyama N, Liu X, Naumann T, et al. Fine-Tuning Large Neural Language Models for Biomedical Natural Language Processing. arXiv 2021;https://arxiv.org/abs/2112.07869.
52. Chen T, Wu M, Li H. A general approach for improving deep learning-based medical relation extraction using a pre-trained model and fine-tuning. Database 2019;2019.
53. PyTorch, AdamW; 2022. Last accessed: December 5th, 2022. https://pytorch.org/docs/stable/generated/torch.optim.AdamW.html.
54. Atanasova P, Simonsen JG, Lioma C, Augenstein I. A Diagnostic Study of Explainability Techniques for Text Classification. arXiv 2020;https://arxiv.org/abs/2009.13295.
55. Zeng C, Evans JP, Faraone JN, Qu P, Zheng YM, Saif L, et al. Neutralization of SARS-CoV-2 variants of concern harboring Q677H. Mbio 2021;12(5):e02510–21.
56. Cheng L, Song S, Zhou B, Ge X, Yu J, Zhang M, et al. Impact of the N501Y substitution of SARS-CoV-2 Spike on neutralizing monoclonal antibodies targeting diverse epitopes. Virology journal 2021;18(1):1–6.
57. Escalera A, Gonzalez-Reiche AS, Aslam S, Mena I, Laporte M, Pearl RL, et al. Mutations in SARS-CoV-2 variants of concern link to increased spike cleavage and virus transmission. Cell host & microbe 2022;30(3):373–387.
58. Raghu D, Hamill P, Banaji A, McLaren A, Hsu YT. Assessment of the binding interactions of SARS-CoV-2 spike glycoprotein variants. Journal of pharmaceutical analysis 2022;12(1):58–64.
59. Cheng MH, Krieger JM, Banerjee A, Xiang Y, Kaynak B, Shi Y, et al. Impact of new variants on SARS-CoV-2 infectivity and neutralization: A molecular assessment of the alterations in the spike-host protein interactions. Iscience 2022;25(3):103939.
60. Kim JM, Rhee JE, Yoo M, Kim HM, Lee NJ, Woo SH, et al. Increase in Viral Load in Patients With SARS-CoV-2 Delta Variant Infection in the Republic of Korea. Frontiers in Microbiology 2022;13.

61. Pohl MO, Busnadiego I, Kufner V, Glas I, Karakus U, Schmutz S, et al. SARS-CoV-2 variants reveal features critical for replication in primary human cells. PLoS biology 2021;19(3):e3001006.
62. Bernasconi A, Gulino A, Alfonsi T, Canakoglu A, Pinoli P, Sandionigi A, et al. VirusViz: Comparative analysis and effective visualization of viral nucleotide and amino acid variants. Nucleic Acids Research 2021;49(15):e90.
63. Cilibrasi L, Pinoli P, Bernasconi A, Canakoglu A, Chiara M, Ceri S. ViruClust: direct comparison of SARS-CoV-2 genomes and genetic variants in space and time. Bioinformatics 2022;38(7):1988–1994.
64. Chen C, Nadeau S, Yared M, Voinov P, Xie N, Roemer C, et al. CoV-Spectrum: analysis of globally shared SARS-CoV-2 data to identify and characterize new variants. Bioinformatics 2022;38(6):1735–1737.
65. Gangavarapu K, Latif AA, Mullen JL, Alkuzweny M, Hufbauer E, Tsueng G, et al. Outbreak. info genomic reports: scalable and dynamic surveillance of SARS-CoV-2 variants and mutations. medRxiv 2022;https://doi.org/10.1101/2022.01.27.22269965.

Click here to access/download;Figure;4panelsCompletePanels.pdf

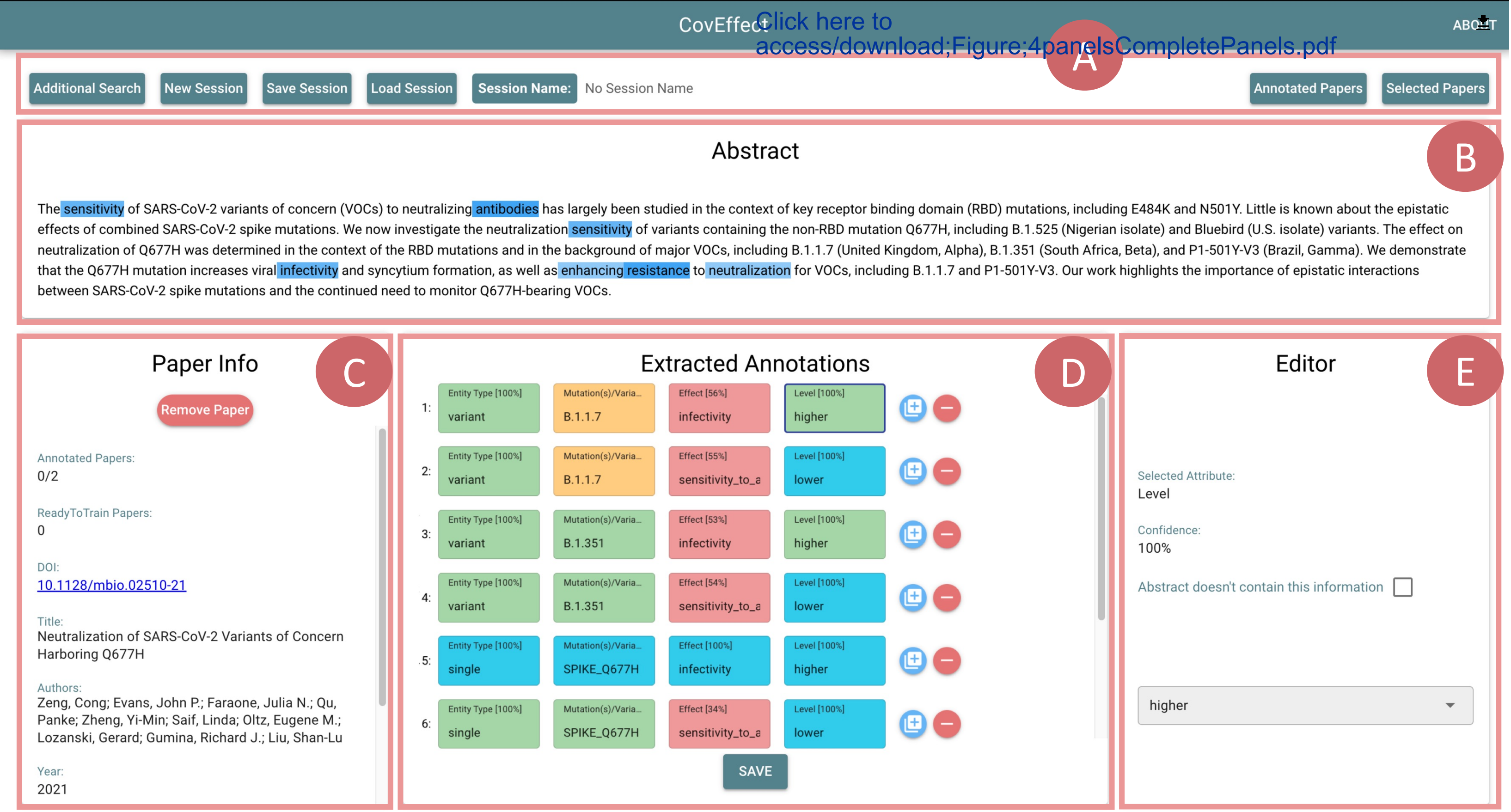

Click here to access/download;Figure;4steps-

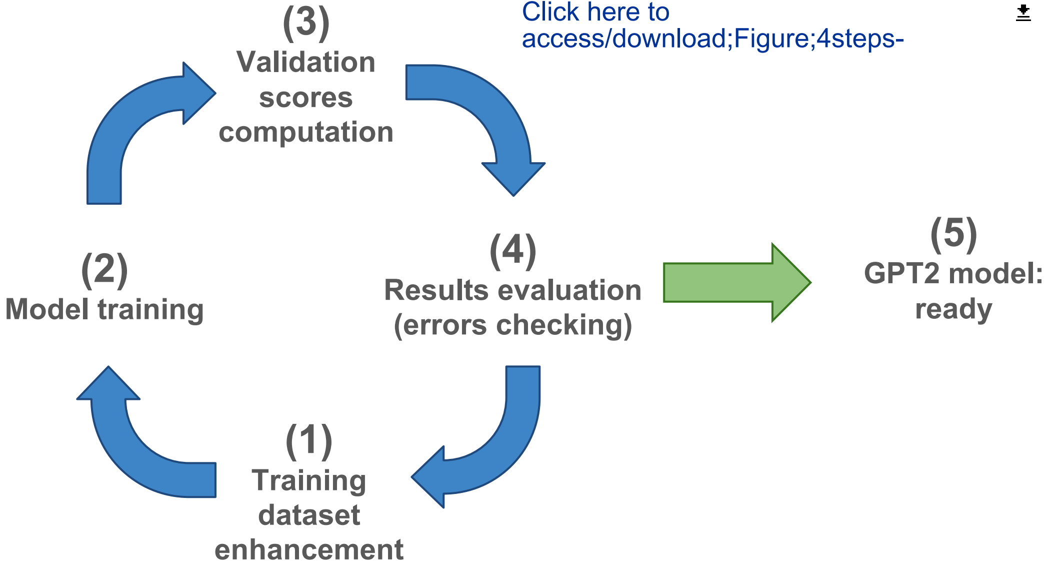

Click here to access/download;Figure;KeywordSearch.pdf

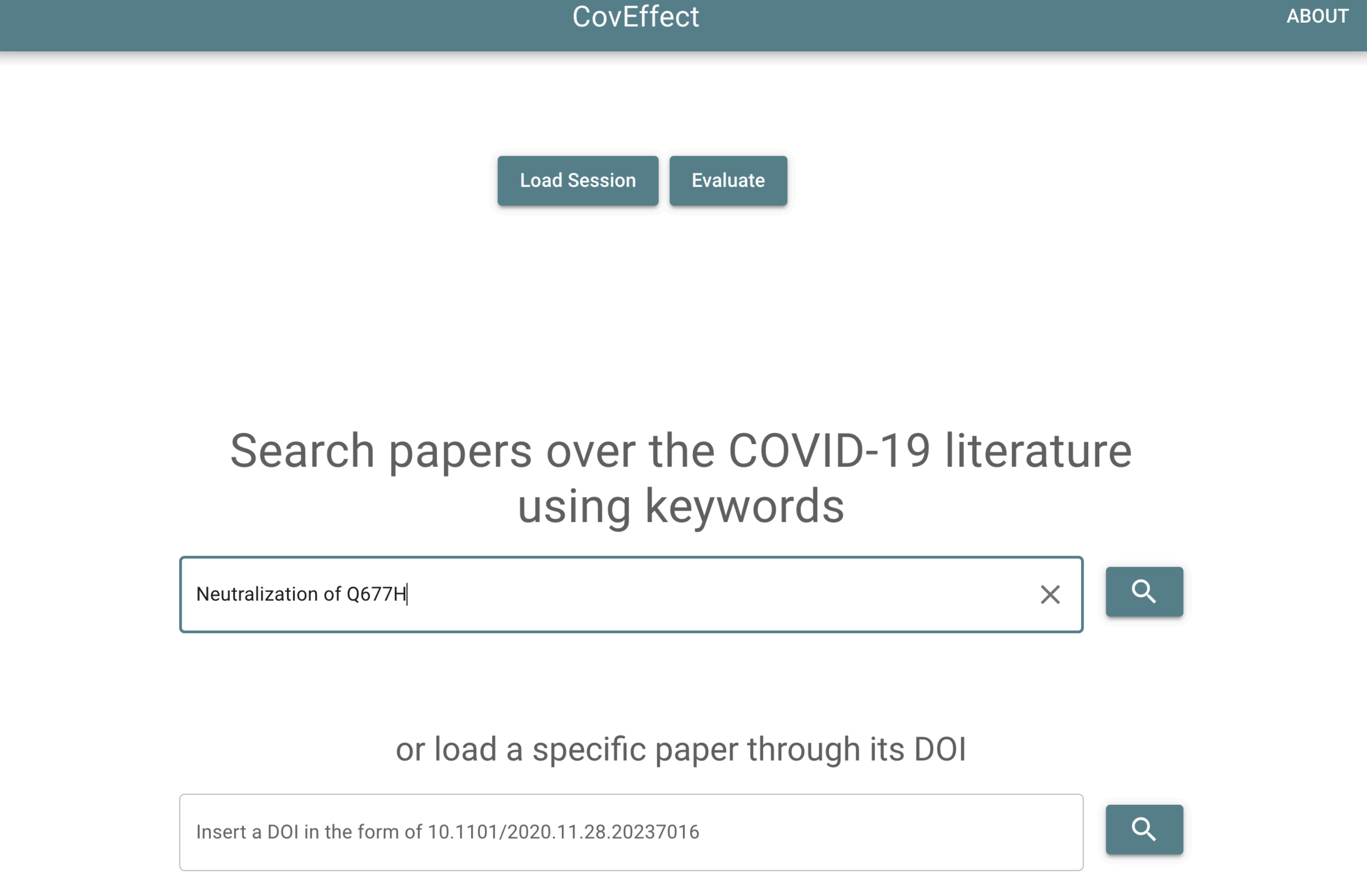

Select relevant papers among the results to continue

## Results for : "Neutralization of q677h"

+ Export TSV

| ☐ | # | Doi | Title | Year | Authors | Abstract | Source | Citations | | Similar To |
|---|---|---|---|---|---|---|---|---|---|---|
| ☑ | 0 | 10.1128/mbio.02510-21 | Neutralization of SARS-CoV-2 Variants of Concern Harboring Q677H | 2021 | Zeng, Cong; Evans, John P.; Faraone, Julia N.; Qu, Panke; Zheng, Yi-Min; Saif, Linda; Oltz, Eugene M.; Lozanski, Gerard; Gumina, Richard J.; Liu, Shan-Lu | The sensitivity of SARS-CoV-2 variants of concern (VOCs) to neutralizing antibodies has largely been studied in the context of key receptor binding domain (RBD) mutations, including E484K and N501Y. Little is known about the epistatic effects of combined SARS-CoV-2 spike mutations. We now investigate the neutralization sensitivity of variants containing the non-RBD mutation Q677H, including B.1.525 (Nigerian isolate) and Bluebird (U.S. isolate) variants. The effect on neutralization of Q677H was determined in the context of the RBD mutations and in the background of major VOCs, including B.1.1.7 (United Kingdom, Alpha), B.1.351 (South Africa, Beta), and P1-501Y-V3 (Brazil, Gamma). We demonstrate that the Q677H mutation increases viral infectivity and syncytium formation, as well as enhancing resistance to neutralization for VOCs, including B.1.1.7 and P1-501Y-V3. Our work highlights the importance of epistatic interactions between SARS-CoV-2 spike mutations and the continued need to monitor Q677H-bearing VOCs. | mBio | 20 | FIND SIMILAR PAPERS | |
| ☑ | 1 | 10.1186/s12985-021-01554-8 | Impact of the N501Y substitution of SARS-CoV-2 Spike on neutralizing monoclonal antibodies targeting diverse epitopes | | Cheng, Lin; Song, Shuo; Zhou, Bing; Ge, Xiangyang; Yu, Jiazhen; Zhang, Mingxia; Ju, Bin; Zhang, Zheng | The emergence and rapid spread of the B.1.1.7 lineage (VOC-202012/01) SARS-CoV-2 variant has aroused global concern. The N501Y substitution is the only mutation in the interface between the RBD of B.1.1.7 and ACE2, raising concerns that its recognition by neutralizing antibodies may be affected. Here, we assessed the neutralizing activity and binding affinity of a panel of 12 monoclonal antibodies against the wild type and N501Y mutant SARS-CoV-2 pseudovirus and RBD protein, respectively. We found that the neutralization activity and binding affinity of most detected antibodies (10 out of 12) were unaffected, although the N501Y substitution decreased the neutralizing and binding activities of CB6 and increased that of BD-23. These findings could be of value in the development of therapeutic antibodies. SUPPLEMENTARY INFORMATION: The online version contains supplementary material available at 10.1186/s12985-021-01554-8. | Virology journal | 23 | FIND SIMILAR PAPERS | 10.1128/mbio.02510-21 |
| ☐ | 2 | 10.26355/eurrev_202109_26805 | Variants of SARS-CoV-2, their effects on infection, transmission and neutralization by vaccine-induced antibodies | 2021 | Wahid, M; Jawed, A; Mandal, R K; Dailah, H G; Janahi, E M; Dhama, K; Somvanshi, P; Haque, S | OBJECTIVE: The current study reviewed Severe Acute Respiratory Syndrome Coronavirus-2 (SARS-CoV-2) variants for their effects on infection, transmission and neutralization by vaccine-induced antibodies. MATERIALS AND METHODS: The research articles for the current study were searched over PubMed, Google Scholar, EMBASE and Web of Science online databases. The keywords used were: (("SARS-CoV-2" OR "COVID-19") AND ("mutation" OR "variant") AND ("death" OR "hospitalization" OR "infection" OR "transmission") AND ("antibody" OR "neutralize" OR "vaccine")). A total of 333 research articles were retrieved through online-database search. These articles were further scrutinized for their relevancy. Additionally, searches were performed to find the latest relevant information over Google search engine and relevant news browsers. Finally, around 35 germane articles were considered for scripting the current report. RESULTS: The mutations have changed amino acids at key positions in spike protein viz. S477N, E484K, Q677H, E484Q, L452R, K417T, K417N and N501Y. These mutations are relevant for different characteristics and are present in newly evolved strains of SARS-CoV-2 like E484K in B.1.526, B.1.525, P.2, B.1.1.7, P.1 and B.1.351. Mutations have increased the immune escape potential leading to 3.5-6.5-folds decrease in neutralization of antibodies (Pfizer and Moderna vaccines). The variant, B.1.617 circulating in India and many other countries (double variant) having E484Q and L452R mutations, has raised the infection rate and decreased the neutralization capacity of the vaccine-induced antibodies. Deadly K417N+E484K+N501Y triplet mutations found in B.1.351 and P.1 have increased the transmission ability of these strains by 50% leading to greater COVID-19 hospitalization, ICU admissions and deaths. CONCLUSIONS: The new SARS-CoV-2 variants have compromised the neutralization potential of the currently used vaccines, but still, they have considerable efficacy to reduce infection and mortality. Graphical Abstract: https://www.europeanreview.org/wp/wp-content/uploads/Graphical_Abstract.jpg. | Eur Rev Med Pharmacol Sci | 8 | FIND SIMILAR PAPERS | |
| ☐ | 3 | 10.1007/s11262-021-01826-z | Isolation and genetic characterization of SARS-CoV-2 from Indian patients in a [illegible] | 2021 | Shrivastava, Shubham; Patil, Harshad P.; Mhaske, Suhas T.; Palkar, Sonali; Lalwani, Sanjay; [illegible] | In view of the rapidly progressing COVID-19 pandemic, our aim was to isolate and characterize SARS-CoV-2 from Indian patients. SARS-CoV-2 was isolated from nasopharyngeal swabs collected from the two members of a family without any history of (H/O) travel abroad. Both the virus isolates (8003 and 8004) showed CPE on day 3 post-inoculation, viral antigens by immunofluorescence assay and produced distinct, clear and uniform plaques. Infectious virus titers were 5 × 10(6) and 4 × 10(6) Pfu/ml by plaque assay and 10(7.5) and 10(7) by CPE-based TCID50/ml, respectively. Phylogenetic analysis grouped our isolates with the Italian strains. On comparison with Wuhan strain, 3 unique mutations were identified in nsp3 (A1812D), exonuclease (P1821S) of Orf1ab and spike protein (Q677H) regions, [illegible] | Virus Genes | 5 | FIND SIMILAR PAPERS | |

2 records selected.

1-4 of 4

Click here to access/download;Figure;pipelineAll5.png

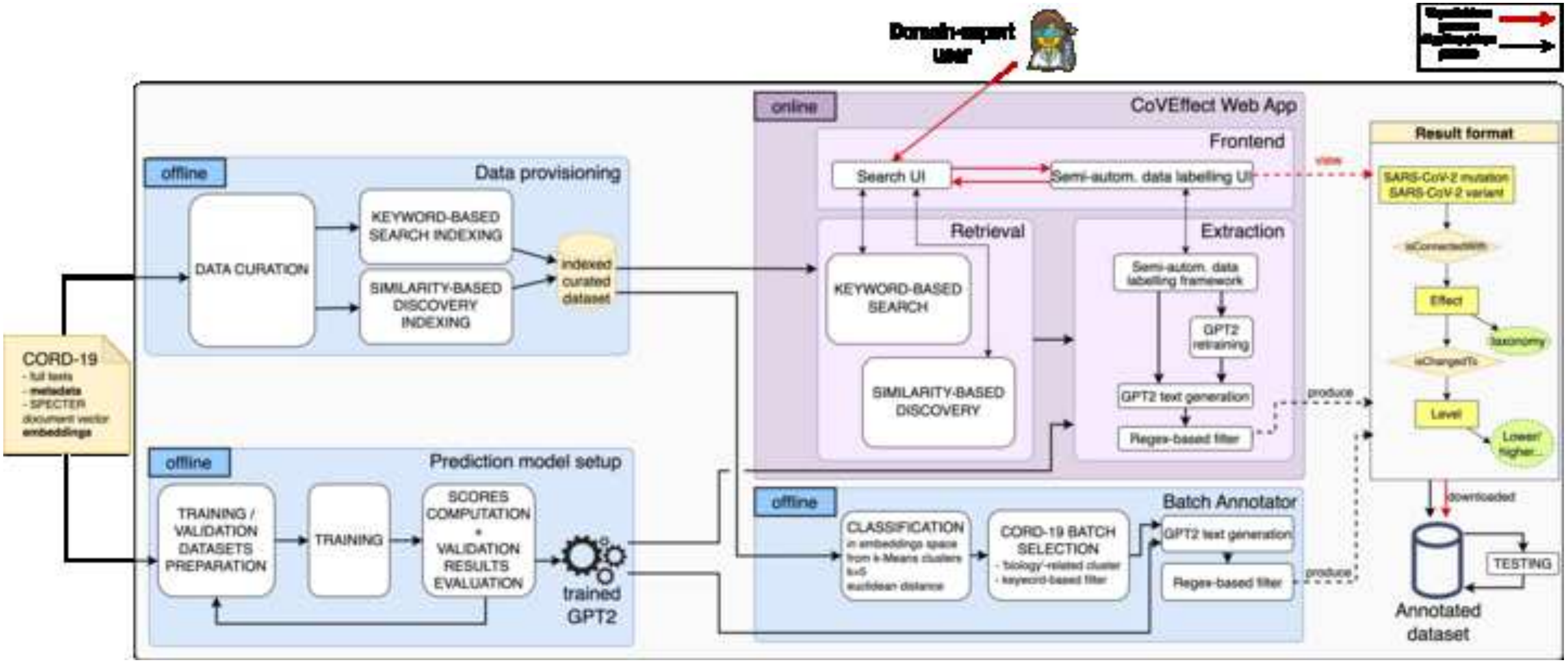

Click here to access/download;Figure;RunningExample.pdf

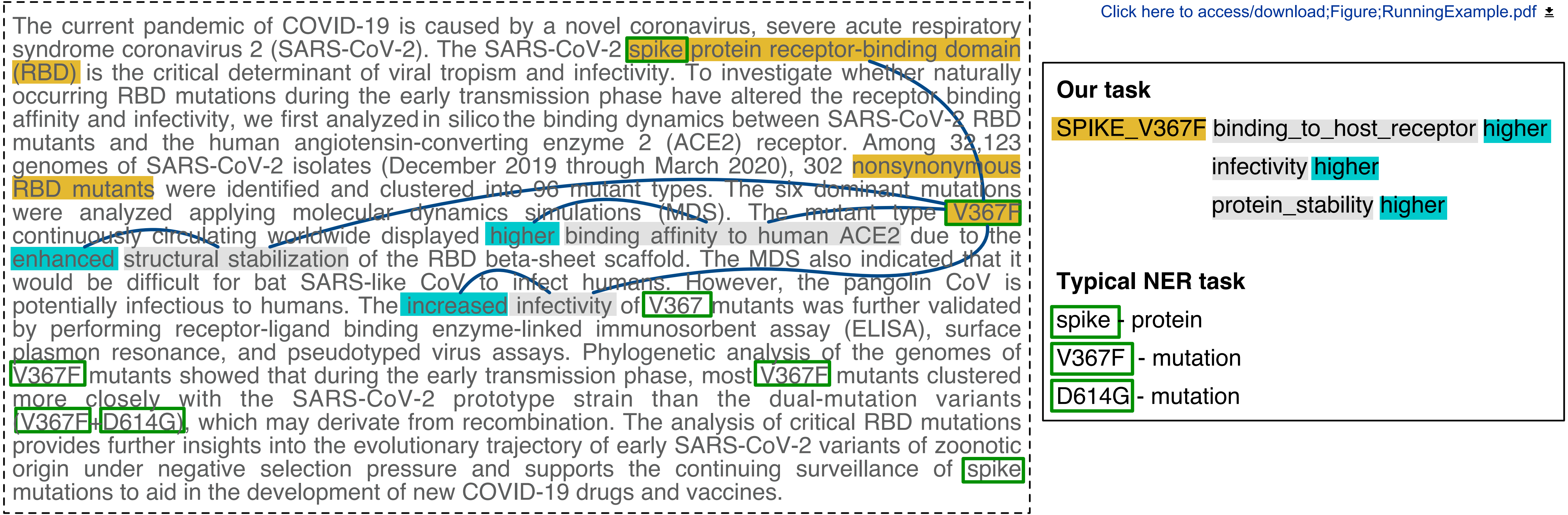

Click here to access/download;Figure;saliencyMapInfV367F.pdf

# Abstract

The current pandemic of COVID-19 is caused by a novel coronavirus, severe acute respiratory syndrome coronavirus 2 (SARS-CoV-2). The SARS-CoV-2 spike protein receptor-binding domain (RBD) is the critical determinant of viral tropism and infectivity. To investigate whether naturally occurring RBD mutations during the early transmission phase have altered the receptor binding affinity and infectivity, we first analyzed in silico the binding dynamics between SARS-CoV-2 RBD mutants and the human angiotensin-converting enzyme 2 (ACE2) receptor. Among 32,123 genomes of SARS-CoV-2 isolates (December 2019 through March 2020), 302 nonsynonymous RBD mutants were identified and clustered into 96 mutant types. The six dominant mutations were analyzed applying molecular dynamics simulations (MDS). The mutant type V367F continuously circulating worldwide displayed higher binding affinity to human ACE2 due to the enhanced structural stabilization of the RBD beta-sheet scaffold. The MDS also indicated that it would be difficult for bat SARS-like CoV to infect humans. However, the pangolin CoV is potentially infectious to humans. The increased infectivity of V367 mutants was further validated by performing receptor-ligand binding enzyme-linked immunosorbent assay (ELISA), surface plasmon resonance, and pseudotyped virus assays. Phylogenetic analysis of the genomes of V367F mutants showed that during the early transmission phase, most V367F mutants clustered more closely with the SARS-CoV-2 prototype strain than the dual-mutation variants (V367F+D614G), which may derivate from recombination. The analysis of critical RBD mutations provides further insights into the evolutionary trajectory of early SARS-CoV-2 variants of zoonotic origin under negative selection pressure and supports the continuing surveillance of spike mutations to aid in the development of new COVID-19 drugs and vaccines.

IMPORTANCE A novel coronavirus, severe acute respiratory syndrome coronavirus 2 (SARS-CoV-2), has caused the pandemic of COVID-19. The origin of SARS-CoV-2 was associated with zoonotic infections. The spike protein receptor-binding domain (RBD) is identified as the critical determinant of viral tropism and infectivity. Thus, whether mutations in the RBD of the circulating SARS-CoV-2 isolates have altered the receptor binding affinity and made them more infectious has been the research hot spot. Given that SARS-CoV-2 is a novel coronavirus, the significance of our research is in identifying and validating the RBD mutant types emerging during the early transmission phase and increasing human angiotensin-converting enzyme 2 (ACE2) receptor binding affinity and infectivity. Our study provides insights into the evolutionary trajectory of early SARS-CoV-2 variants of zoonotic origin. The continuing surveillance of RBD mutations with increased human ACE2 affinity in human or other animals is critical to the development of new COVID-19 drugs and vaccines against these variants during the sustained COVID-19 pandemic.

Click here to access/download;Figure;threesteps.png

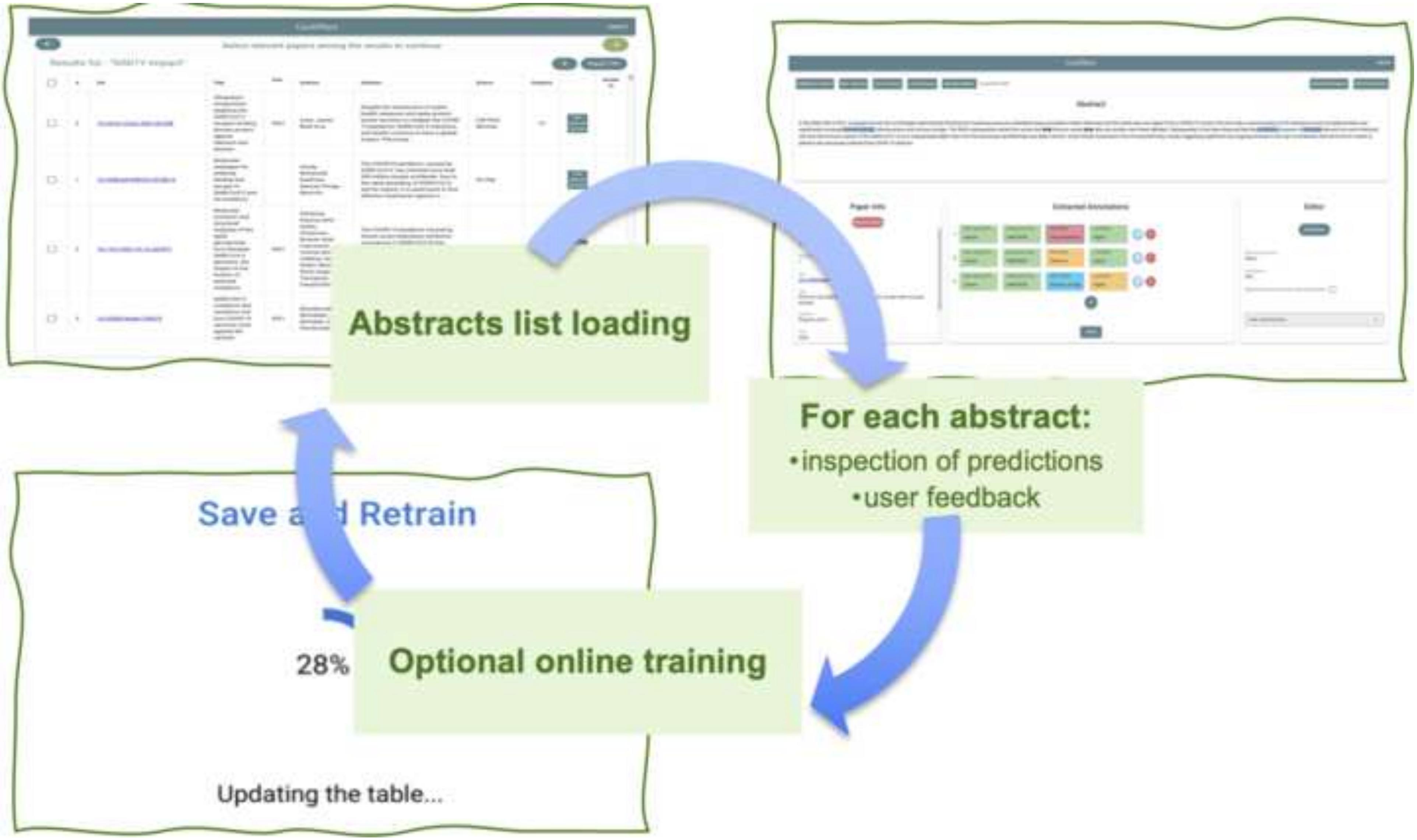

Click here to access/download;Figure;UML_activity_diagram.png

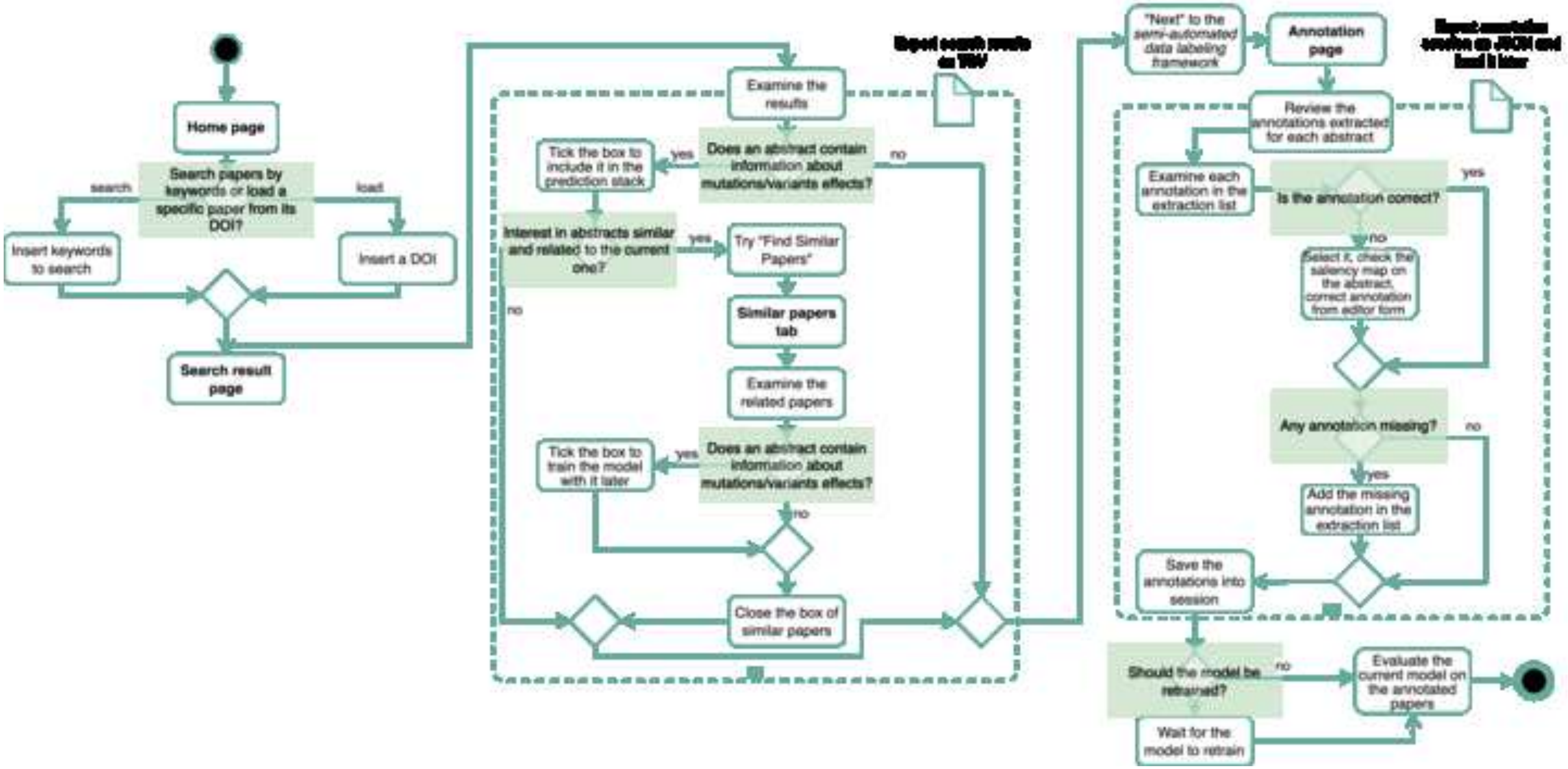

Cover Letter
Click here to access/download;Personal Cover;cover_letter_gigascience_coveffect.pdf

**DIPARTIMENTO DI ELETTRONICA, INFORMAZIONE E BIOINGEGNERIA**
**POLITECNICO DI MILANO**
Via Ponzio 34/5 - I-20133 Milano – Italy

Anna Bernasconi – e.mail: anna.bernasconi@polimi.it
Tel. (+39.02)23993655

December 5th, 2022

To: Scott Edmunds
Editor-in-Chief, GigaScience

Dear Professor Edmunds,

Find enclosed the paper "***CoVEffect: Interactive Deep Learning-based SARS-CoV-2 Mutations and Variants Effect Extraction***", by Giuseppe Serna Garcia, Ruba Al Khalaf, Francesco Invernici, Stefano Ceri, and myself, hereby submitted to the GigaScience journal.

Recently, we witnessed the generation of enormous amounts of literature describing the SARS-CoV-2 virus and connected information. An important corpus of COVID-19-related articles was collected in the CORD-19 project and is still largely unexplored. One type of knowledge that can be extracted, refers to the effects of specific mutations and variants on the phenotype of the virus and on the disease it elicits. These effects may include aspects related to immunology, epidemiology, viral kinetics, and disease treatment.

To this end, we developed a complete framework that allows us to 1) retrieve abstracts of interest from CORD-19 and 2) recognize in the text information related to mutations or variants effects, thereby producing structured tuples that can be usefully integrated within data pipelines on mutations and variants.
The predictions are obtained by exploiting a Natural Language Processing transformer-based model (i.e., GPT2), trained to generate tuples from abstracts that describe the connection between a given mutation or variant, its effect, and how this has changed with respect to the wild-type of the virus.
Predictions can be operated on a batch of abstracts (offline) or on single abstracts selected by an expert user on the CoVEffect Web application (http://gmql.eu/coveffect/), which assists expert users with proposed predicted annotations and the possibility to correct or add annotations. The new annotations can be used to re-train the current model and improve its performance.
The overall framework can be easily adapted to resolve similar unstructured-to-structured text translation tasks, which are typical of biomedical domains.

Our group has recently dedicated considerable efforts to the genomic data integration problem, and we are continuously looking for solutions to support this process with automation. In this respect, see the GeMI system (https://doi.org/10.1093/database/baac036) used to extract key-value attribute pairs from the experiment and sample descriptions from the Gene Expression Omnibus.

All listed authors have participated actively in the project, have read, and approved the submitted version of the manuscript, and are prepared to take public responsibility for its contents. The authors also guarantee that the content of the submitted manuscript has not been published or submitted for publication elsewhere. For any questions regarding our submission, please do not hesitate to contact us.

With my best regards,

Anna Bernasconi
Assistant Professor
Department of Electronics, Information, and Bioengineering
Politecnico di Milano
