# Supplementary material for: CoVEffect: interactive system for mining the effects of SARS-CoV-2 mutations and variants based on deep learning
Source: Gigascience. 2023 May 23;12:giad036. doi: 10.1093/gigascience/giad036 (PMC10205000; doi:10.1093/gigascience/giad036)
Supplement: giad036_Response_to_Reviewer_Comments_Original_Submission [file giad036_response_to_reviewer_comments_original_submission.pdf]

# Author's Response To Reviewer Comments

Close

Please kindly refer to the response_to_reviewers.pdf file, attached as Supplementary Material. We preferred to provide a PDF rather than plain text to provide a clearer text with formatting, color, and a table.

Close
