# Supplementary material for: CoVEffect: interactive system for mining the effects of SARS-CoV-2 mutations and variants based on deep learning
Source: Gigascience. 2023 May 23;12:giad036. doi: 10.1093/gigascience/giad036 (PMC10205000; doi:10.1093/gigascience/giad036)
Supplement: giad036_Reviewer_1_Report_Original_Submission — Bahrad Sokhansanj -- 1/30/2023 Reviewed [file giad036_reviewer_1_report_original_submission.pdf]

Reviewer Report

Title: CoVEffect: Interactive System for Mining the Effects of SARS-CoV-2 Mutations and Variants Based on Deep Learning

Version: Original Submission **Date:** 1/30/2023

Reviewer name: Bahrad Sokhansanj

Reviewer Comments to Author:

I was not aware that the review process was going to be public; accordingly, I will keep my comments brief. I do believe that the paper is in large part suitable for publication. It is well-written and presented. It addresses an important problem, which is extracting information about SARS-CoV-2 variants from a vast body of literature that has been produced since the onset of the pandemic. This is necessarily for further progress in developing models relating genomic variants to impacts on COVID-19 disease severity, immune evasion, and therapeutic resistance. For example, in our work we have sought to rely on GISAID database entries accompanying sequence submissions that have metadata from publications and unpublished reports, but the data are presented in a non-systematic way and are likely incomplete. (See, e.g., our recent papers at https://pubmed.ncbi.nlm.nih.gov/36041271/ and https://pubmed.ncbi.nlm.nih.gov/36552295/.

The principal scientific contribution of the paper, in my view, is the carefully curated and annotated database and the provision of an interface and open source for the community to utilize. I note that the use of GPT-2 and saliency maps for explainability are not themselves novel more generally (nor do the authors claim that is the case). The validation of these approaches against experts is helpful. However, I do believe that the method should be placed in a better context. The only paper cited for work prior to transformers appears to be a 2018 review that was not published in an open-access journal. Reviews and papers cited for background context should be open access to the extent possible. If the only review were not open access, then a selection of individual open-access works should be cited. However, I am certain that there must be open-access reviews out there (and I was not particularly impressed by the abstract of the one that the authors did cite). I also strongly urge the authors to benchmark their methods, to the extent possible, against a non-transformer based approach such as that of Singhal et al. (https://pubmed.ncbi.nlm.nih.gov/27902695/). If that is not appropriate for this problem, the authors should explain why. I understand that the authors claim that named-entity methods are perhaps not necessarily suitable for the entity extraction task, but I'm not necessarily certain that's the case. And, if so, why would the extraction problem necessarily depend on the transformer being used rather than a more traditional approach. Quantitative results would help make that case.

Methods

Are the methods appropriate to the aims of the study, are they well described, and are necessary controls included? Choose an item.

Conclusions

Are the conclusions adequately supported by the data shown? Choose an item.

Reporting Standards

Does the manuscript adhere to the journal's guidelines on minimum standards of reporting? Choose an item.

Choose an item.

Statistics

Are you able to assess all statistics in the manuscript, including the appropriateness of statistical tests used? Choose an item.

Quality of Written English

Please indicate the quality of language in the manuscript: Choose an item.

Declaration of Competing Interests

Please complete a declaration of competing interests, considering the following questions:

- Have you in the past five years received reimbursements, fees, funding, or salary from an organisation that may in any way gain or lose financially from the publication of this manuscript, either now or in the future?
- Do you hold any stocks or shares in an organisation that may in any way gain or lose financially from the publication of this manuscript, either now or in the future?
- Do you hold or are you currently applying for any patents relating to the content of the manuscript?
- Have you received reimbursements, fees, funding, or salary from an organization that holds or has applied for patents relating to the content of the manuscript?
- Do you have any other financial competing interests?
- Do you have any non-financial competing interests in relation to this paper?

If you can answer no to all of the above, write 'I declare that I have no competing interests' below. If your reply is yes to any, please give details below.

I declare that I have no competing interests.

I agree to the open peer review policy of the journal. I understand that my name will be included on my report to the authors and, if the manuscript is accepted for publication, my named report including any attachments I upload will be posted on the website along with the authors' responses. I agree for my report to be made available under an Open Access Creative Commons CC-BY license (http://creativecommons.org/licenses/by/4.0/). I understand that any comments which I do not wish to

be included in my named report can be included as confidential comments to the editors, which will not be published.

Choose an item.

To further support our reviewers, we have joined with Publons, where you can gain additional credit to further highlight your hard work (see: https://publons.com/journal/530/gigascience). On publication of this paper, your review will be automatically added to Publons, you can then choose whether or not to claim your Publons credit. I understand this statement.

Yes Choose an item.
