# Supplementary material for: CoVEffect: interactive system for mining the effects of SARS-CoV-2 mutations and variants based on deep learning
Source: Gigascience. 2023 May 23;12:giad036. doi: 10.1093/gigascience/giad036 (PMC10205000; doi:10.1093/gigascience/giad036)
Supplement: giad036_Reviewer_2_Report_Original_Submission — Theo Sanderson -- 2/14/2023 Reviewed [file giad036_reviewer_2_report_original_submission.pdf]

Reviewer Report

Title: CoVEffect: Interactive System for Mining the Effects of SARS-CoV-2 Mutations and Variants Based on Deep Learning

Version: Original Submission **Date:** 2/14/2023

Reviewer name: Theo Sanderson

Reviewer Comments to Author:

This paper addresses an important issue: while there is a huge literature on COVID-19, a tiny fraction of it is organised in a machine readable form such that it can be used in large scale analyses, for example in combination viral sequencing data. There is a real and pressing need to address these issues. CovEffect proposes to address these issues with two connected contributions: an interface that enables users to manually extract structured annotations from abstracts, and a machine-learning model that attempts to extract these annotations automatically. These two contributions are synergistic in that the ML-model is run on manuscripts before they are presented to the user, enabling a semi-automated approach where the user corrects the model's output with support from saliency maps that show what the model is looking at; and where the model can be retrained on the basis of continually annotated data.
The authors do a good job of characterising the behaviour of the model. It performs imperfectly, with a precision of 0.59 and a recall of 0.44 in terms of predictions of full tuples. In my opinion these results mean this model is perhaps not yet precise enough to gain widespread use in the near future. Similarly, I felt the annotation tool was a proof-of-concept, which is valuable -- with the saliency maps especially interesting -- rather than a tool I could immediately imagine myself using. Nevertheless, I think the ideas and development described in this manuscript are a useful contribution to the community, as are the manually extracted annotations provided which could be used to train future models. The authors usefully describe specific limitations of the model, and how these could be addressed, and make their code available.
I have some comments, which I will go through below. These comments do not question the authors' ability to demonstrate the claims that they make in the paper, and in general I think work to address them would be out of scope. I do not request that the authors do substantial work to address these comments, and think the paper provided is already a useful contribution.

____

Bigger ideas
The idea of representing mutations and their effects as tuples of (variant/mutation, defined_category_of_effect, directionality_of_effect) is a useful abstraction. Something that this doesn't model that will potentially be important for the future is the more complex interaction of genetic background with individual mutations. This has been well demonstrated for SARS-CoV-2 and so tuples of "background, mutation, [the rest]" may be important in the future.
In my opinion a key feature for improving the predictive performance of this model will be to start with a different model: either a generally larger model such as GPT-3, which could probably be finetuned relatively easily on this existing data, but has downsides in terms of not being open-source, making the

saliency approach difficult, or a small model more specialised biological data. BioGPT-large would likely be a good open-source choice, which while not as large as GPT-3 has specialised domain knowledge in biology, and excels at answering questions from PubMed abstracts. This model was not released as this paper was being developed so was not an option available here. I am not requesting this change for this manuscript.
"Long cycle training" section - it would be good to clarify if this type of training always starts from scratch with the initial weights from hugging-face, or not.
Minor wording points (optional)
"unprecedented attention of the research community towards the mechanisms of the virus" => this reads a bit awkwardly because it can sound like it's about SARS-CoV-2 being more studied than before the pandemic, but it wasn't studied before the pandemic. Could say "has made the SARS-CoV-2 virus one of the most studied viruses in the world, with research on.." or similar.
Grammar: "Allowed to target" => "allowed the targeting of" or better "has addressed several..."
"We use GPT2 to learn tuples" -- better to say "extract"/"predict"
Grammar "are either only supporting" => "each support only one of"
"requires to connect" => "requires connecting"
"EXplainable AI" => explainable AI
"duple contribution" => two contributions
"payed off" => paid off
"Unclear result presentation.s" => "Unclear result presentations"
"could exempt from declaring" => "might not declare"

Methods

Are the methods appropriate to the aims of the study, are they well described, and are necessary controls included? Choose an item.

Conclusions

Are the conclusions adequately supported by the data shown? Choose an item.

Reporting Standards

Does the manuscript adhere to the journal's guidelines on minimum standards of reporting? Choose an item.

Choose an item.

Statistics

Are you able to assess all statistics in the manuscript, including the appropriateness of statistical tests used? Choose an item.

Quality of Written English

Please indicate the quality of language in the manuscript: Choose an item.

Declaration of Competing Interests

Please complete a declaration of competing interests, considering the following questions:

- Have you in the past five years received reimbursements, fees, funding, or salary from an organisation that may in any way gain or lose financially from the publication of this manuscript, either now or in the future?
- Do you hold any stocks or shares in an organisation that may in any way gain or lose financially from the publication of this manuscript, either now or in the future?
- Do you hold or are you currently applying for any patents relating to the content of the manuscript?
- Have you received reimbursements, fees, funding, or salary from an organization that holds or has applied for patents relating to the content of the manuscript?
- Do you have any other financial competing interests?
- Do you have any non-financial competing interests in relation to this paper?

If you can answer no to all of the above, write 'I declare that I have no competing interests' below. If your reply is yes to any, please give details below.

I declare that I have no competing interests

I agree to the open peer review policy of the journal. I understand that my name will be included on my report to the authors and, if the manuscript is accepted for publication, my named report including any attachments I upload will be posted on the website along with the authors' responses. I agree for my report to be made available under an Open Access Creative Commons CC-BY license (http://creativecommons.org/licenses/by/4.0/). I understand that any comments which I do not wish to be included in my named report can be included as confidential comments to the editors, which will not be published.

Choose an item.

To further support our reviewers, we have joined with Publons, where you can gain additional credit to further highlight your hard work (see: https://publons.com/journal/530/gigascience). On publication of this paper, your review will be automatically added to Publons, you can then choose whether or not to claim your Publons credit. I understand this statement.

Yes Choose an item.
